# Covert neural and autonomic signatures of shared perception

**DOI:** 10.1093/scan/nsag009

**Published:** 2026-02-17

**Authors:** Mustafa Yavuz, Jamal Esmaily, Bahador Bahrami, Ophelia Deroy

**Affiliations:** Graduate School of Systemic Neurosciences, Ludwig-Maximilians-Universität München, Munich, 82152, Germany; Faculty of General and Experimental Psychology, Ludwig-Maximilians-Universität München, Munich, 80802, Germany; Cognition, Values and Behavior Research Group, Ludwig-Maximilians-Universität München, Munich, 80333, Germany; Graduate School of Systemic Neurosciences, Ludwig-Maximilians-Universität München, Munich, 82152, Germany; Faculty of General and Experimental Psychology, Ludwig-Maximilians-Universität München, Munich, 80802, Germany; Faculty of General and Experimental Psychology, Ludwig-Maximilians-Universität München, Munich, 80802, Germany; Cognition, Values and Behavior Research Group, Ludwig-Maximilians-Universität München, Munich, 80333, Germany; Chair of Philosophy of Mind, Faculty of Philosophy, Philosophy of Science and Religious Studies Ludwig-Maximilians-Universität München, Munich, 80539, Germany; Munich Center for Neurosciences, Ludwig-Maximilians-Universität München, Munich, D-82152, Germany; Institute of Philosophy, School of Advanced Study, University of London, London, WC1E 7HU, United Kingdom

**Keywords:** social vigilance, social influence, perception, decision-making, autonomic response

## Abstract

Humans often co-perceive stimuli with others, yet the neurocognitive effects of such shared perceptual contexts are underexplored. We tested whether awareness that a visual stimulus is simultaneously available to another person, without interaction, modulates behavioral performance and neurophysiological signatures of perceptual decision-making. Thirty-three participants completed 640 trials of a Random Dot Kinematogram motion discrimination task while EEG and pupillometry data were recorded. A confederate was present, with a divider ensuring that, on each trial, the stimulus was either jointly visible to both or privately visible to the participant. Participants received no feedback and engaged in no interaction, isolating the effect of joint visibility. Behavioral performance was unaffected by social context, but EEG analysis revealed context-specific neural patterns emerging after cue onset and before stimulus presentation, suggesting proactive encoding of the social context. Additionally, pupil size was significantly greater during public visibility trials, indicating heightened arousal associated with social vigilance. These findings suggest that co-perception induces covert social vigilance—anticipatory arousal and neural readiness in response to co-visibility, even without interaction. Such covert markers could serve as biomarkers for altered social salience processing in clinical populations, such as those with social anxiety disorder or autism.

## Introduction

The idea that social factors—such as the judgments or mere presence of others—can affect perception is not new. Triplett (1898) showed that cyclists performed better when racing others than alone, an effect termed *social facilitation*. This early finding inspired later research on how social presence shapes task performance. By the mid-20th century, the “New Look” movement emphasized the influence of social context on cognitive functions, including perception (Bruner 1992). Pioneers like Asch (1956), Sherif (1935), and Milgram (1963) demonstrated that groups could sway perceptual reports. Zajonc (1965) argued that others’ presence increases arousal, enhancing performance on well-learned tasks but hindering new learning—a distinction between social facilitation and social inhibition. Beyond changes in performance or reports under observation (Park et al. 2023), recent work investigates how social factors directly influence perceptual processes (e.g. Germar et al. 2014, Otten et al. 2017, Zanesco et al. 2019a, Kampis and Southgate 2020, Scheller and Sui 2022a, 2022b, Deroy et al. 2024).

Building on evidence that joint attention modulates perception (Seow and Fleming 2019, Wahn et al. 2020, Battich et al. 2021), we asked an even simpler question: does mere awareness that others perceive the same thing change our own perception? In daily life—on public transport, in a cinema, or reading a book—we intuitively distinguish between shared and private visual experiences. Crucially, this awareness of co-perception does not require coordination, interaction, or gaze cues (Samson et al. 2010, Polosan et al. 2011, Vesper et al. 2016, McKay et al. 2021, Pisauro et al. 2022, Stephenson et al. 2021, Philippe et al. 2024).

This *minimal sensitivity to co-perception* (Deroy et al. 2024) is ubiquitous, but how is it represented in the brain? Chein et al. (2011) found that peer presence heightened adolescent—but not adult—risk-taking and ventral striatum activity, suggesting amplified reward sensitivity under observation. van Hoorn et al. (2018) extended this by showing that in adults, social presence instead activated evaluative and self-referential regions, notably the medial prefrontal cortex.

Further insights come from studies on social evaluative pressure. Anticipating judgment by observers triggers stress-related neural and hormonal responses, including activation of the amygdala and anterior cingulate cortex, increased cortisol, heart rate, and pupil size (Goodman et al. 2017, Fehlner et al. 2020). Cottrell et al. (1968) proposed that mere presence influences behavior only when evaluation is expected—a view supported by later studies (Henchy and Glass 1968, Paulus and Murdoch 1971, Cohen 1980).

Sanders (1981) identified three mechanisms through which others’ presence might affect brain and behavior: reflexive arousal, motivational modulation, and attentional conflict. Neural evidence since has supported all three to varying degrees. Yet, these early theories largely ignored the neural impact of *mere presence* in the absence of overt behavior. In contrast, our study focuses on underlying brain processes triggered by others’ presence, independent of motivation, evaluation, or behavioral change. This approach aligns with recent consciousness research, where covert neural and ocular markers are recognized as valid indicators of awareness (Schiff et al. 2007, Kondziella et al. 2020, Edlow et al. 2023), and complements studies of implicit social perception (Swe et al. 2020, Oomen et al. 2022).

We test the hypothesis that co-perception triggers *social vigilance*—a heightened readiness prompted by the possibility that others may act upon or coordinate around the same objects we perceive. Crucially, we use the term social vigilance to refer to an anticipatory coding of social relevance: a pre-stimulus context representation (here, public vs. private) that configures neural states and global arousal to prioritize socially relevant processing. This usage is distinct from evaluative apprehension (Kirschbaum et al. 1992); in our design, co-visibility was cued but explicit feedback was absent precluding formal evaluation. Hence, we use “social vigilance” in this precise, anticipatory sense. Similarly, it is also distinct from neighboring concepts such as social facilitation (Zajonc 1965), evaluation apprehension (Cottrell et al. 1968), and joint attention (Tomasello et al. 2005); none of which are required by our minimal co-visibility setup. Nor does co-perception entail implicit mentalizing or uncertainty monitoring; rather, it labels a cue-triggered task-state representation that configures attention and arousal before sensory evidence arrives.

In our usage, social vigilance is a top-down and predictive (Rao and Ballard 1999, Clark 2013) pre-stimulus configuration of neural states and arousal that prepares processing for upcoming potential social input. This view aligns with accounts of social perception and communication as mutual inference under predictive coding, where internal models guide top-down configuration even before evidence arrives (Friston and Frith 2015). Here and throughout, we use co-perception for the phenomenon—the awareness that another is co-perceiving the same object or event without requiring coordination, gaze-cueing, or mind-reading—and we interpret the present neural and pupillometric findings as social vigilance, an anticipatory mechanism through which co-perception has the potential to bias processing (cf. Deroy et al. 2024).

To isolate and empirically test the co-perception effects from audience effects and coordination-related (e.g. joint attention) confounds, we developed a new paradigm in which participants sat beside a confederate, with visual stimuli displayed in either shared or private screen regions. This design modulates co-visibility while holding physical co-presence constant, akin to sitting next to someone on a bus who may or may not see your screen (Hamilton and Lind 2016, Oh et al. 2018). Using a non-spatial task, it avoids automatic perspective taking (Samson et al. 2010, Seow and Fleming 2019). With this pre-registered design (https://osf.io/yjafh/), we examine whether awareness of co-perception—without interaction—modulates perception, decision-making, metacognitive efficiency, EEG responses, and pupil dilation. Rather than focusing solely on behavior, we ask whether brain and eye data reveal covert signatures of social awareness, employing a decoding-based approach increasingly used to detect latent mental states (King and Dehaene 2014, Haynes 2009).

## Materials and methods

### Participants

We conducted a pilot study (N = 9; 4 females) with a similar setup (see Supplementary Material) to determine sample size. Based on preregistered power analysis we recruited 34 right-handed adults (mean age = 23.88, SD = 4.39; 11 males). One participant was excluded for below-chance warm-up performance. Due to technical issues, we excluded pupillometry data from four participants and one block of behavioral data from one participant. All had normal or corrected-to-normal vision, no neurological or psychiatric history, and received €25 compensation. Ethical approval was obtained from the LMU Munich Psychology Faculty Ethics Committee (No: 7_4_18052020).

### Display, stimuli, and response

**Figure 1.**
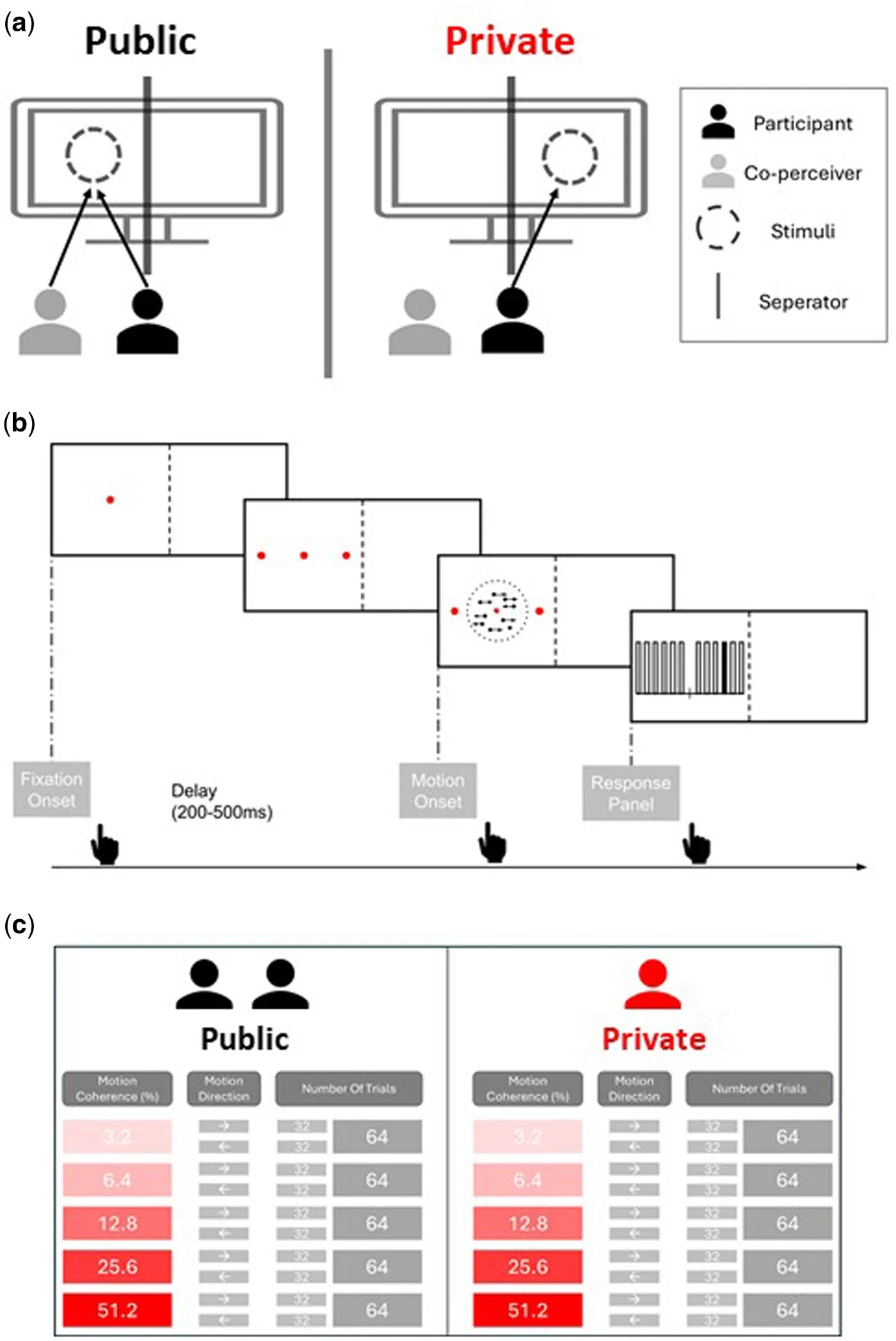
Experimental design. (a) Overview of the experimental setup. A cardboard physical separator (gray straight line) divided the display into two compartments. In each trial, the stimuli appeared in one of the two compartments of the screen. Private and public conditions were implemented by having the confederate (gray) sit to one side of the participant (black). (b) Sequence of events in a trial. (c) Overview of the trial types and corresponding number of trials in a given session.

To contrast co-perception, a black cardboard (25 × 50 cm) divided the display vertically, ensuring only the near half was shared with a seated confederate (same individual across participants, Fig. 1a). This setup made one side of the screen *public* and the other *private*, depending on confederate position. Each block (four total) randomized confederate seating (two left, two right), ensuring balanced stimulus-public/private mapping. Participants were familiarized with the setup to confirm understanding of visual access constraints.

Perceptual decisions were assessed using a free-response Random Dot Kinematogram (RDK) task (Shadlen and Newsome 2001) implemented in Psychtoolbox-3 (Brainard 1997). Participants indicated the average motion direction (left/right) of dots at 5 coherence levels (3.2–51.2%). Each trial began with a fixation dot, followed by the motion stimulus after a random delay (200–500 ms). On each frame, ∼17.8 white dots (0.03°) moved within an 8° invisible circle. Participants had up to 5000 ms to respond by moving the mouse sideways (≥1° visual angle), which terminated the stimulus and revealed a response panel with 12 vertical bars (6 per side). They clicked a bar on the chosen side to report confidence (1–6), with distance from the center indicating confidence level (Fig. 1b). This integrated choice-confidence report followed prior studies (Bahrami et al. 2012, Bang et al. 2017, Esmaily et al. 2023). Trials were separated by 1000 ms. Sessions were held in dim lighting with ∼70 cm viewing distance. Stimuli were shown on an HP ZR30W LCD monitor (29.7”, 60 Hz, 2560 × 1600).

### Experimental design

The experiment manipulated three factors: Motion Coherence (five levels), Motion Direction (left/right), and Social Context (private/public), yielding 20 unique trial types. Each participant completed 32 trials per type, totaling 640 trials across four blocks (160 trials per block), with breaks between blocks (Fig. 1c).

### Procedure

Participants began with a 160-trial warm-up block without the confederate. Afterward, average accuracy was shown; if it exceeded chance, the experiment continued; otherwise, the session was terminated. EEG and pupillometry preparations followed. Once EEG setup was complete, the confederate was introduced and the main task began. At session end, participants completed two debriefing questionnaires (see Supplementary Material).

### Behavioral measures and analysis

We recorded binary choices (left/right), reaction times (ms), and confidence ratings (1: Not sure at all to 6: Completely sure). Metacognitive accuracy was calculated by applying Receiver Operating Characteristic (ROC) analysis and computing Area Under the Curve (AUC) for each condition as described in Fleming (2017).

### Psychometric curve fitting

To model accuracy as a function of stimulus coherence, we fitted a two-parameter Weibull function for each participant and condition (public vs. private):


P(x) = 1 - (1/2) *  exp(-(x/λ)κ)

where *P*(*x*) is the probability of a correct response at coherence *x*, λ (threshold) indicates the coherence level at which accuracy reaches ∼82%, and κ (slope) reflects sensitivity. Parameters were estimated using nonlinear least squares regression (nlinfit, MATLAB 2018b), with initial values λ  =  25 and κ  =  3. Threshold and slope values were used to compare perceptual decision-making across conditions.

### EEG recordings and preprocessing

EEG was recorded using a 64-channel EasyCap system and BrainVision amplifier at 1000 Hz. Electrodes followed the 10–20 system; impedance was kept below 25 kΩ. TP7 and TP11, placed under the collarbones, recorded ECG and were referenced offline to yield a single trace. EEG preprocessing used EEGLAB v2022.0 (Delorme and Makeig 2004) and custom MATLAB scripts. A notch filter (45–55 Hz) removed power noise, followed by a bandpass filter (0.1–100 Hz). Epochs were extracted around (1) Motion onset and (2) Response (−3000 to +6000 ms). Noisy channels were visually identified and interpolated using spherical spline (mean = 0.76 channels per block, ∼1%, SD = 1.36). Artifact-contaminated epochs were manually rejected (mean = 24.58 trials per subject, ∼4%, SD = 16.29). Data was re-referenced to the average (excluding ECG). Independent Component Analysis (“runica”) removed eye-movement artifacts. On average, 1.23 components (2%, SD = 0.48) were removed using ICLabel, and cleaned EEG was reconstructed.

### Pupillometry recordings and preprocessing

Pupil size changes were recorded with PupilLab’s “Pupil Core” eye-tracker (Kassner et al. 2014) at 200 Hz. The ambient light conditions of the experimental room were kept constant across participants, and the luminance of the screen between public and private trials were kept the same during the sessions. This was achieved by having the same side of the screen, i.e. the same physical stimulus on the screen, serve as the public or private condition depending on the spatial positioning of the confederate who was seated behind and to the side of the participant. Raw diameter and confidence scores were exported from Pupil Player as CSV files. Preprocessing was conducted using custom Python scripts. Data were filtered to retain only samples detected with the “pye3d 0.3.0 real-time” method. The first 5 seconds of each recording were excluded to eliminate artifacts from display luminance changes (e.g. software initialization). Pupil diameters <1.0 mm or >7.0 mm were discarded, as were samples with confidence ≤0.99. Missing values were interpolated using backward filling.

Blinks were identified using external blink onset/offset timestamps, and the corresponding data were removed and interpolated. Left and right eye data were merged: the inter-eye diameter difference was computed and interpolated; missing values for one eye were estimated based on this difference; and the mean diameter across eyes was calculated per time point. Physiologically implausible rapid changes were removed by computing dilation speed (absolute derivative) and applying a median absolute deviation (MAD)-based threshold; exceeding values were excluded and interpolated.

The cleaned signal was smoothed using a fourth-order low-pass Butterworth filter (4 Hz cutoff). Data were segmented into 9-second epochs aligned to stimulus onset (6 s baseline, 3 s post-onset), interpolated, and resampled onto a 200 Hz uniform time grid. These preprocessed epochs were used for further analysis. For the response-locked analysis, as pre-registered, we quantified mean pupil size in the 1 s interval immediately preceding the behavioral response, using the 500 ms interval directly preceding this window as the baseline. For the stimulus-locked analysis, pupil traces were baseline-corrected using the −1000 to −500 ms interval before motion onset.

### ERP analysis

Preprocessed EEG data was used for event-related potentials (ERPs) analysis via custom written MATLAB scripts. In order to calculate amplitude of the centro-parietal positivity (CPP), we followed a similar procedure to Kelly and O’Connel (2013) and Vafaei Shooshtari et al. (2019). Stimulus-locked EEG traces from the CPz electrode were baseline corrected (baseline period: from −200 ms until motion onset). Then, per each trial, we computed the mean CPP amplitude between 400 and 600 ms post stimulus-onset time period. A similar procedure was applied for response-locked ERP analysis. Preprocessed EEG traces from CPz were baseline corrected relative to response onset (0–200 ms post response window).

### Whole sensor space representational similarity analysis (RSA)

To complement traditional ERP analyses, we conducted a time-resolved multivariate pattern analysis using Representational Similarity Analysis (RSA) (Kriegeskorte et al. 2008, Nili et al. 2014), following Lucykx et al. (2019), to examine how EEG activity encodes (1) motion coherence and (2) social context. This method compared observed neural representational dissimilarity matrices (Brain RDMs) to theoretical model RDMs over time.

For each participant, preprocessed EEG data—containing trial-wise responses across all electrodes and time points—were aligned with behavioral data, where motion coherence (3.2, 6.4, 12.8, 25.6, 51.2%) and social context (public/private) served as predictors. Baseline correction was applied using the −500 to 0 ms window before stimulus onset. EEG signals were z-scored within electrodes and time points for cross-trial comparability.

We then performed multiple linear regression at each electrode and time point, modeling z-scored EEG activity using trial-specific predictors. Condition-specific neural response patterns were extracted from the regression coefficients (excluding the intercept). PCA was applied to the beta weights, and the least significant singular component was removed. At each time point, a Mahalanobis distance matrix was computed between conditions, yielding 10 × 10 symmetric Brain RDMs (5 coherence levels × 2 social contexts).

Two 10 × 10 Model RDMs were constructed. The coherence model RDM encoded absolute coherence differences, normalized by the maximum (51.2), and replicated its 5 × 5 structure across both social contexts. The social context model RDM assigned zero to within-context comparisons and one to between-context comparisons, constructed by repeating a 5 × 5 identity matrix across condition pairs. To quantify similarity between neural and model RDMs, we computed Kendall’s Tau-A rank correlation at each time point, yielding time-resolved RSA scores for each participant. For a schematic figure explaining our RSA pipeline, see Supplementary Figure S11.

### Searchlight representational similarity analysis (RSA)

To localize effects of motion coherence and social context, we applied a searchlight RSA by repeating the same RSA procedure within predefined electrode clusters, enabling rough spatial localization across the sensor space. We defined five electrode ROIs: Frontal (AF7, AF3, AF4, AF8, AFz, F1, F3, F5, F7, F2, F4, F6, F8, Fz), Centro-parietal (CP1, CP2, CP3, CP4, CPz, Pz), Lateral-parietal (P1, P3, P5, P7, P9, P2, P4, P6, P8), Temporal (T7, T8, TP7, TP8), and Occipital (O1, O2, Oz, POz, PO7, PO3, PO4, PO8).

### Statistical testing

Behavioral measures (Accuracy, Reaction Time, Confidence Ratings) were analyzed using linear or generalized linear mixed-effect models in RStudio 2023.06.0 (RStudio Team 2020) via the lme4 package (Bates et al. 2015), depending on outcome distributions. Since ROC curves and AUCs were computed at the subject level, we used paired-samples *t*-tests to examine the effect of social context on metacognitive efficiency. Pre-registered hypotheses on stimulus-locked ERPs and response-locked pupil size were tested with linear mixed-effect models. For the Inter-Trial Interval pupil size hypothesis, we modeled mean pupil size during the 1000 ms post-response window as a function of motion coherence, confidence ratings, and social context (fixed effects), with subjects as random effects. For stimulus-locked pupil size, which lacked a pre-registered hypothesis, we followed an exploratory approach: linear mixed-effect models were fit to each time point in the pupil size time series, yielding coefficients and *P*-values per sample, followed by Benjamini-Hochberg FDR correction (Benjamini and Hochberg 1995).

To assess whether RSA time series differed from zero, we conducted cluster-based permutation tests (Maris and Oostenveld 2007). For each time point, a one-sample *t*-test against zero was run across subjects. Clusters were formed from contiguous time points with *P* < .05, and cluster mass was defined by summing the absolute *t*-values. A null distribution was generated via 1000 permutations by shuffling subject-level RSA time series, recomputing t-tests, and extracting the maximal cluster mass from each iteration. Observed cluster masses were compared to this null distribution, and corrected *P*-values were computed using a bias-adjusted method. Clusters with corrected *P* < .05 were deemed significant. For each significant cluster, we extracted its time range, peak and mean *t*-values, and degrees of freedom. For descriptive purposes only, we then averaged Tau-A values within the (pre-identified) significant temporal clusters for each participant (and, where relevant, within their pre- and post-stimulus portions) and tested these means against zero, reporting the corresponding within-subject effect sizes (Cohen’s *d*). These effect-size estimates are intended to summarize the magnitude of the cluster effects and are not used for additional statistical inference beyond the original cluster-based permutation test.

## Results

### Behavioral results

**Figure 2.**
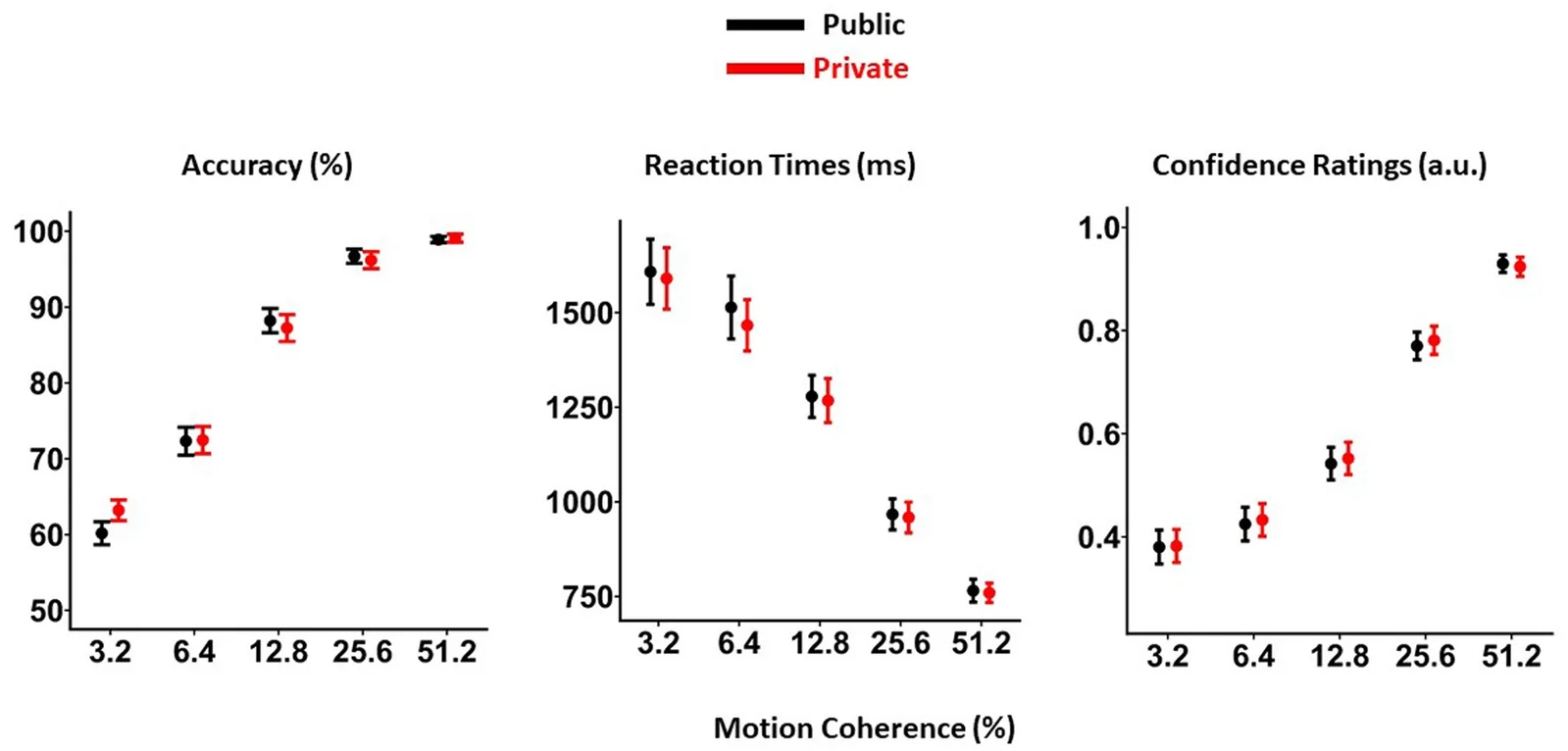
Modulation of accuracy, reaction times, and confidence ratings by motion coherence. Colors indicate social context, and error bars show group-level SEs.

Initially, we compared accuracy, reaction times, and confidence ratings across experimental conditions and coherence levels of motion stimuli. Mixed-effect models showed that accuracy (OR = 1.13, 95% CI [1.12, 1.14], *P* < .001) and confidence ratings (OR = 1.12, 95% CI [1.12, 1.13], *P* < .001) increases with increased coherence, while reaction times (β = −0.01, SE = 0.0002, *t* = −87.5, *P* < .001) decrease. In line with our pre-registered hypotheses, there was no significant effect of social context on accuracy (OR = 1.03, 95% CI [0.95, 1.12], *P* = .43), confidence ratings (OR = 1.06, 95% CI [0.98, 1.15], *P* = 0.11) and reaction times (β=−0.01, SE = 0.006, *t* =−1.73, *P *= .08) (Fig. 2).

**Figure 3.**
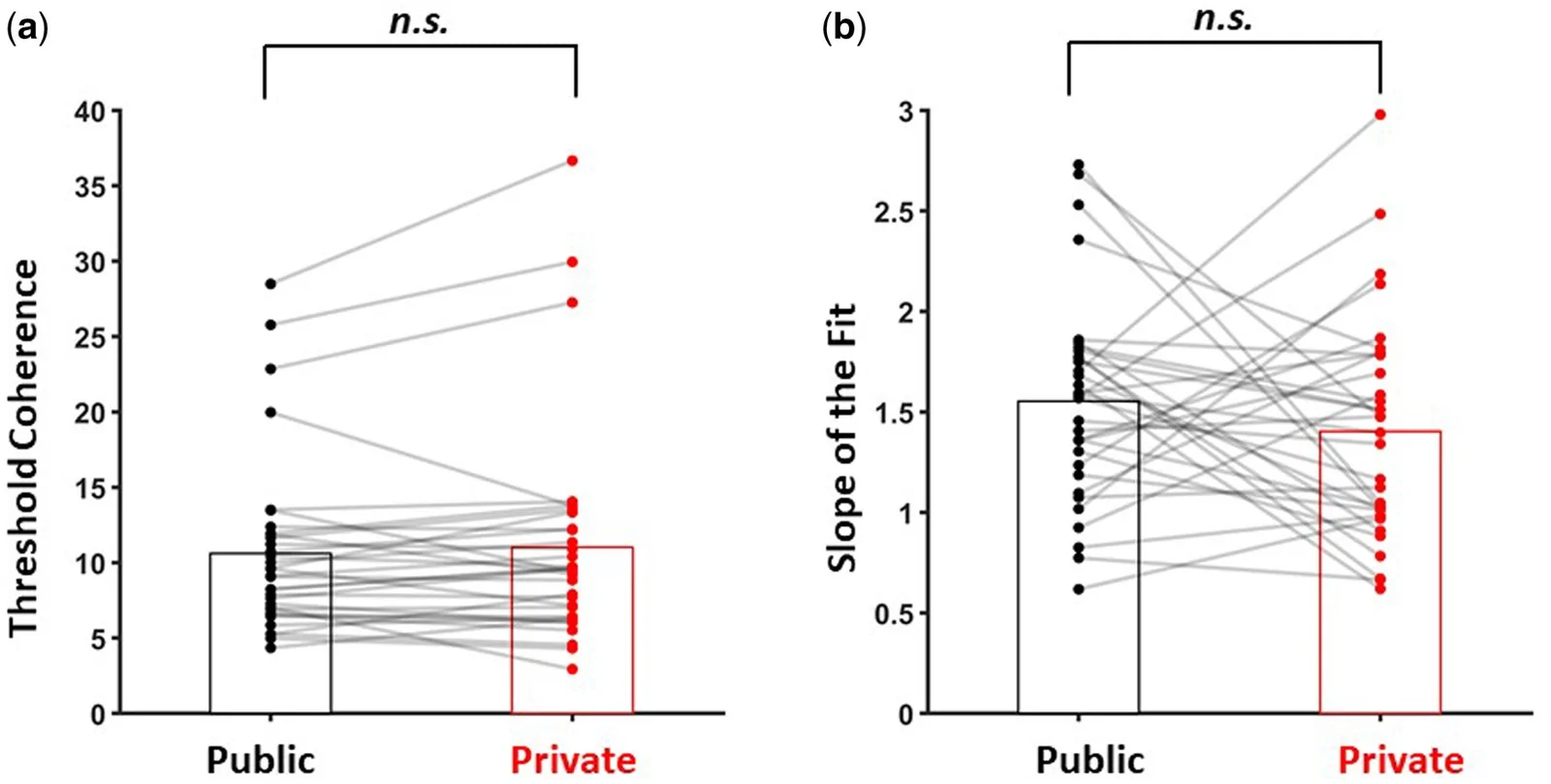
Threshold (a) and slope (b) values obtained from psychometric curves for public and private conditions. Bars represent mean values for each condition, dots show individual subject values.

Finally, the threshold and slope values obtained from psychometric curve fitting were compared between public and private conditions via paired samples *t*-tests. Neither threshold (*t*(32) = −0.87, *P* = .30), nor slope (*t*(32) = 1.17, *P* = .25) differed significantly between conditions (Fig. 3).

**Figure 4.**
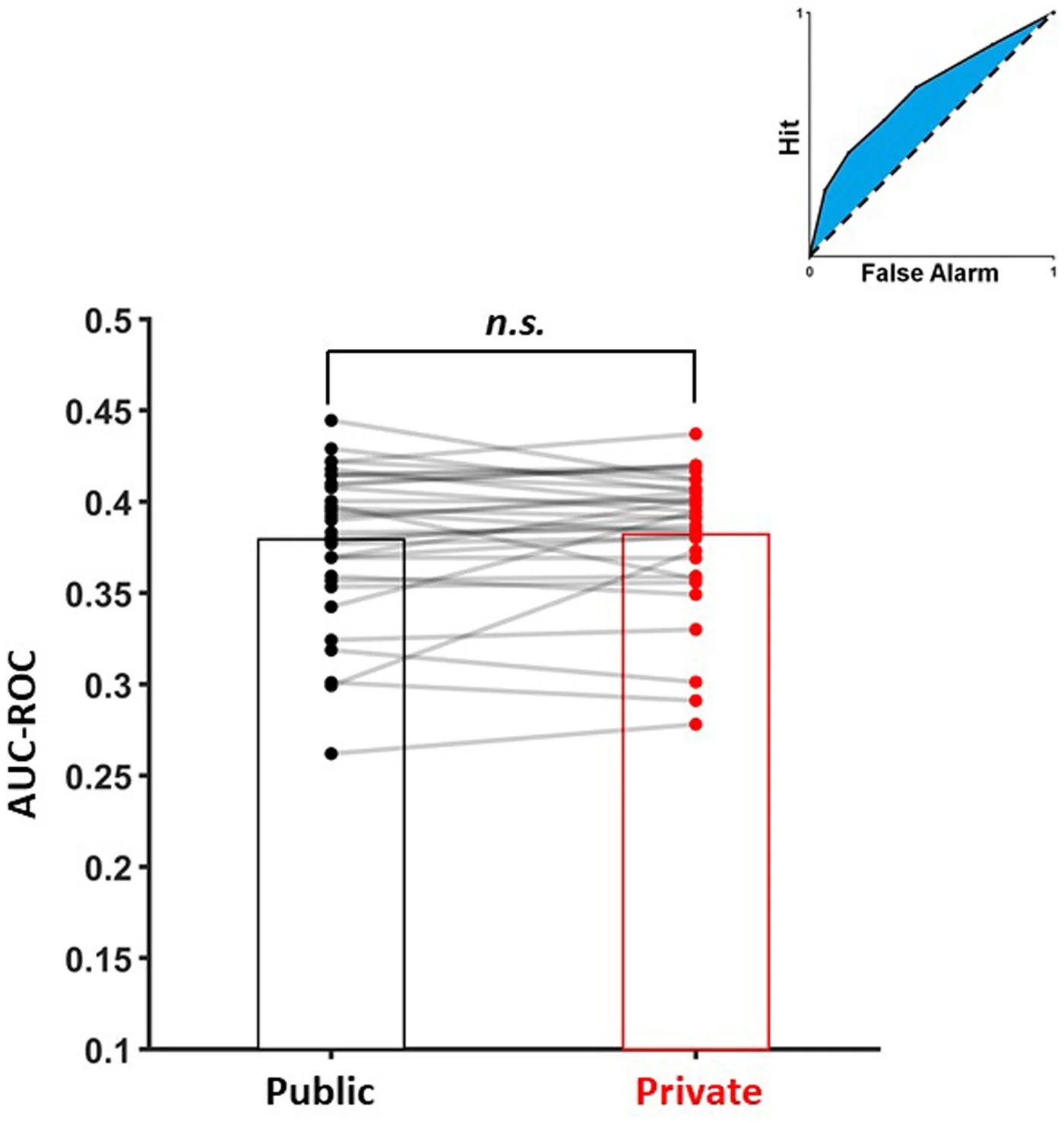
Metacognitive efficiency between experimental conditions, quantified as AUC of ROC fits per subject and condition. Bars represent mean AUC values of ROC fits, per condition. Dots represent AUC values of ROC fits per subject and per condition. The illustration in the top right corner shows what AUCs correspond to relative to false alarms and hits.

**Figure 5.**
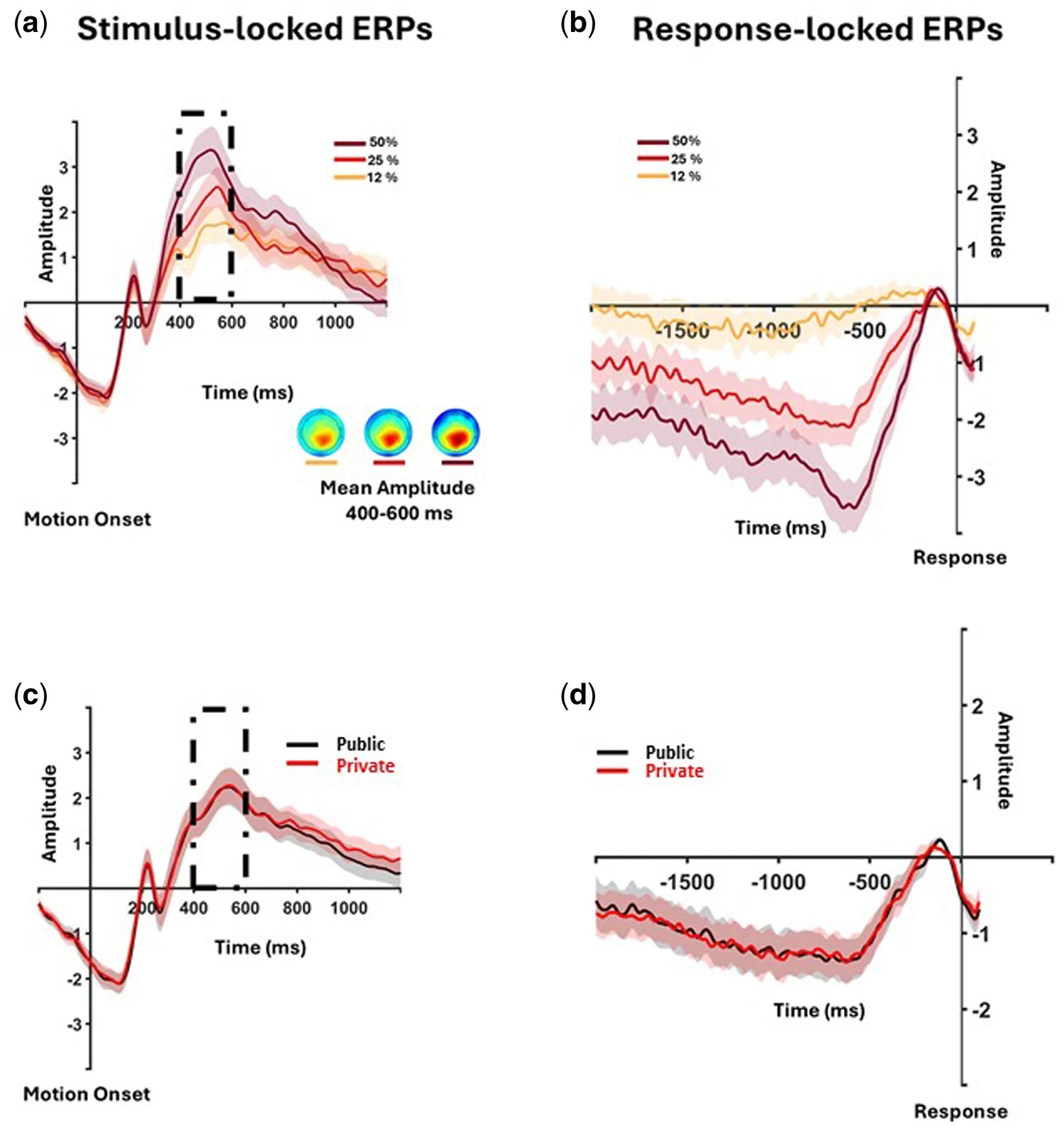
Stimulus- and response-locked EEG modulations by motion coherence and social context. (a, c) Stimulus-locked ERPs at CPz (across participants). The dashed rectangle at 400–600 ms post-motion onset marks the window used to compute mean CPP amplitude for statistical tests. Topographies in A show mean EEG activity for each coherence level, averaged across participants, 400–600 ms post-onset. (b–d) Response-locked signals for motion coherence and social context. Shaded bands denote ±1 SE across participants.

### Metacognitive efficiency

We calculated ROC curves and areas under these curves per each condition and per participants to investigate metacognitive efficiency of our sample during the task. Then, Area Under the Curve (AUC) of these graphs had been calculated to quantify metacognitive efficiency. The result of the *t*-test showed that participants did not show any significant difference between public and private conditions (*t*(32) = −0.71, *P *= 0.48, Fig. 4).

### EEG results

#### Event-related potentials (ERP) analysis

Akin to behavioral analysis, first, we checked to replicate the well-documented relationship between motion coherence and amplitude of the CPP component. Mean CPP amplitude between 400 and 600 ms post-stimulus onset was significantly modulated by motion coherence (β = 0.03, SE = 0.002, *t *= 13.21, *P*<.001, Fig. 5a). However, there wasn’t a significant effect of social context on mean CPP amplitude (β = 0.03, SE = 0.09, *t *= 0.32, *P *= 0.75, Fig. 5c).

### Pupil size results

**Figure 6.**
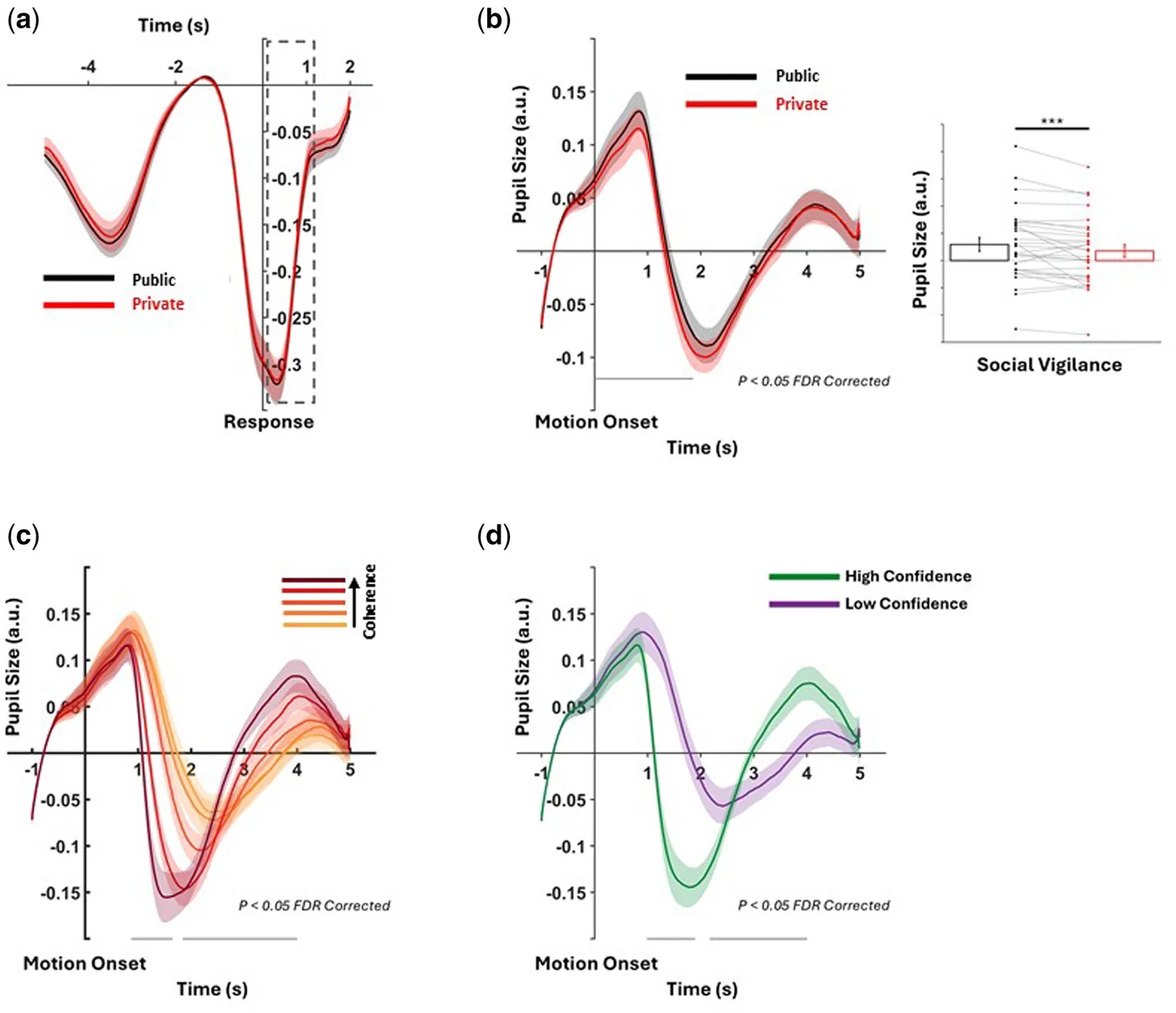
Stimulus- and response-locked pupil-size changes by motion coherence, social context, and confidence. (a) Response-locked pupil change by social context; dashed rectangle marks the analysis window. (b–d) Stimulus-locked pupil responses by social context, motion coherence, and self-reported confidence, respectively. Black horizontal bars indicate windows with FDR-corrected significant regression results (*P* < .05) for each predictor. Shaded bands denote ±1 SE. The summary plot beside B shows mean pupil size during the significant period for the social-vigilance predictor, per subject and condition. Note: Confidence ratings were binned (low/high) only for visualization; statistical analyses used raw ­confidence scores.

First, we tested the hypothesis we had pre-registered regarding the post-decision pupil size and the social context. Our analysis on mean pupil size during the first 1-second period after response did not show any significant difference between private and public perception trials (Fig. 6a), *t*(18104) = 1.15, *β* = 0.004, SE = 0.004, *P* > .05). In contrast, confidence ratings (*t*(18104) = 12.99, *β* = 0.11, SE = 0.008, *P* < .001) and motion coherence (*t*(18104) = 4.85, *β* = 0.004, SE = 0.007, *P* < .001) were significant predictors of the mean pupil size during this time window.

Second, we conducted exploratory analyses on post-stimulus pupil size. As described in the Methods section, we ran a mixed-effects GLM model on post-stimulus onset pupil size data over time (for 4 seconds after motion onset). This regression model showed that motion coherence (Time window 1: 875–1645 ms post-stimulus onset; Time window 2: 1845–4000 ms post-stimulus onset), confidence ratings (Time window 1: 990–1890 ms post-stimulus onset; Time window 2: 2175–4000 ms post-stimulus onset) and social context (Time window: 25–1855 ms post-stimulus onset) are all significant predictors of post-stimulus pupil size. Interestingly, however, the time courses of these effects differ (see Fig. 6). As a complementary summary analysis, we averaged pupil size per trial within the social context-sensitive time window identified by the time-resolved GLM (cf. Fig. 6) and fit a linear mixed model with social context (public vs. private) and motion coherence as fixed effects and a random intercept for subjects. This confirmed a reliable difference between public and private trials: the model-estimated mean difference was 0.0117 (SE = 0.0026), 95% CI [0.0067, 0.0168]; *t*(18,278) = 4.56, *P* < .001 (Fig. 6b). Yet, the standardized effect was rather small, Cohen’s *d* = 0.067, 95% CI [0.039, 0.097], obtained via parametric bootstrap with 1000 refits of the mixed model.

### Exploratory EEG analysis

#### Whole sensor space representational similarity analysis (RSA)

**Figure 7.**
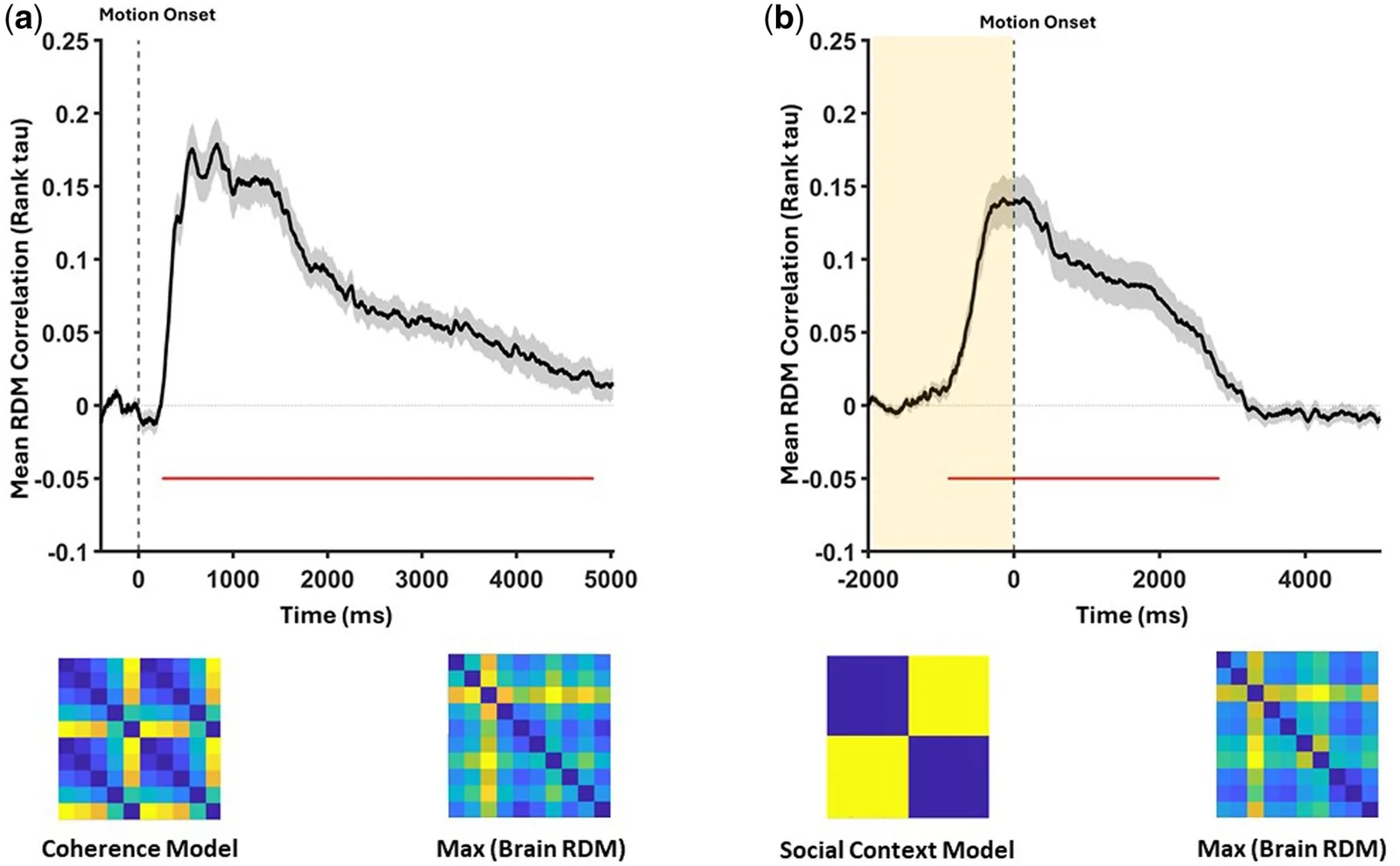
Rank–tau correlations over time between model and neural RDMs. (a, b) Time-series correlations for the coherence and social-context model RDMs with neural RDMs, respectively. Gray dashed vertical lines mark motion onset; red horizontal lines mark significant temporal clusters. In b, the shaded rectangle indicates the pre-stimulus window where social-context representation emerged. Bottom row: the coherence and social-context model RDMs alongside the neural RDMs at each model’s peak correlation.

In order to assess whether the social context is differentially represented in the brain signals across trial time, we applied RSA on the EEG dataset. As described in the methods, we built two model RDMs (see Fig. 7), one for representation of motion coherence and another for representation of social context. Results of the RSA analysis showed that both motion coherence and social context were represented in EEG signals, independently of each other. Interestingly, social context representation started before the onset of motion. Rather, the ramping activity of social context representation was found to be temporally corresponding to the onset of fixation on the screen (i.e. the marker also signifies whether a given trial is going to be publicly co-perceived or private and individually perceived). Our cluster-based permutation analysis revealed one significant temporal cluster for social context representation (Cluster Mass = 10264, *t*(32) = 5.56, *P* < .001, time range: −916 to 2772 ms, relative to motion onset). The analysis of coherence representation also showed 7 significant temporal clusters (largest: Cluster Mass = 13921, *t*(32) = 10.57, *P* < .001, time range: 258–4642 ms). Details of other significant clusters can be found in the Supplementary Material. To quantify the magnitude of these effects, we computed within-subject averages of Tau-A within the relevant cluster windows. For the social-context model, the pre-stimulus portion of the significant cluster (−916–0 ms) showed a mean Tau-A of 0.09 (SD = 0.07), *t*(32) = 7.73, *P* < .001, Cohen’s *d* = 1.35, 95% CI [0.07, 0.12]. The post-stimulus portion of the same cluster (0–2772 ms) yielded a mean Tau-A of 0.08 (SD = 0.09), *t*(32) = 5.71, *P* < .001, Cohen’s *d* = 0.99, 95% CI [0.05, 0.11]. For the coherence model, mean Tau-A within the post-stimulus window (285–4642 ms) was 0.10 (SD = 0.06), *t*(32) = 9.97, *P* < .001, Cohen’s *d* = 1.74, 95% CI [0.08, 0.12].

#### Searchlight representational similarity analysis (RSA)

**Figure 8.**
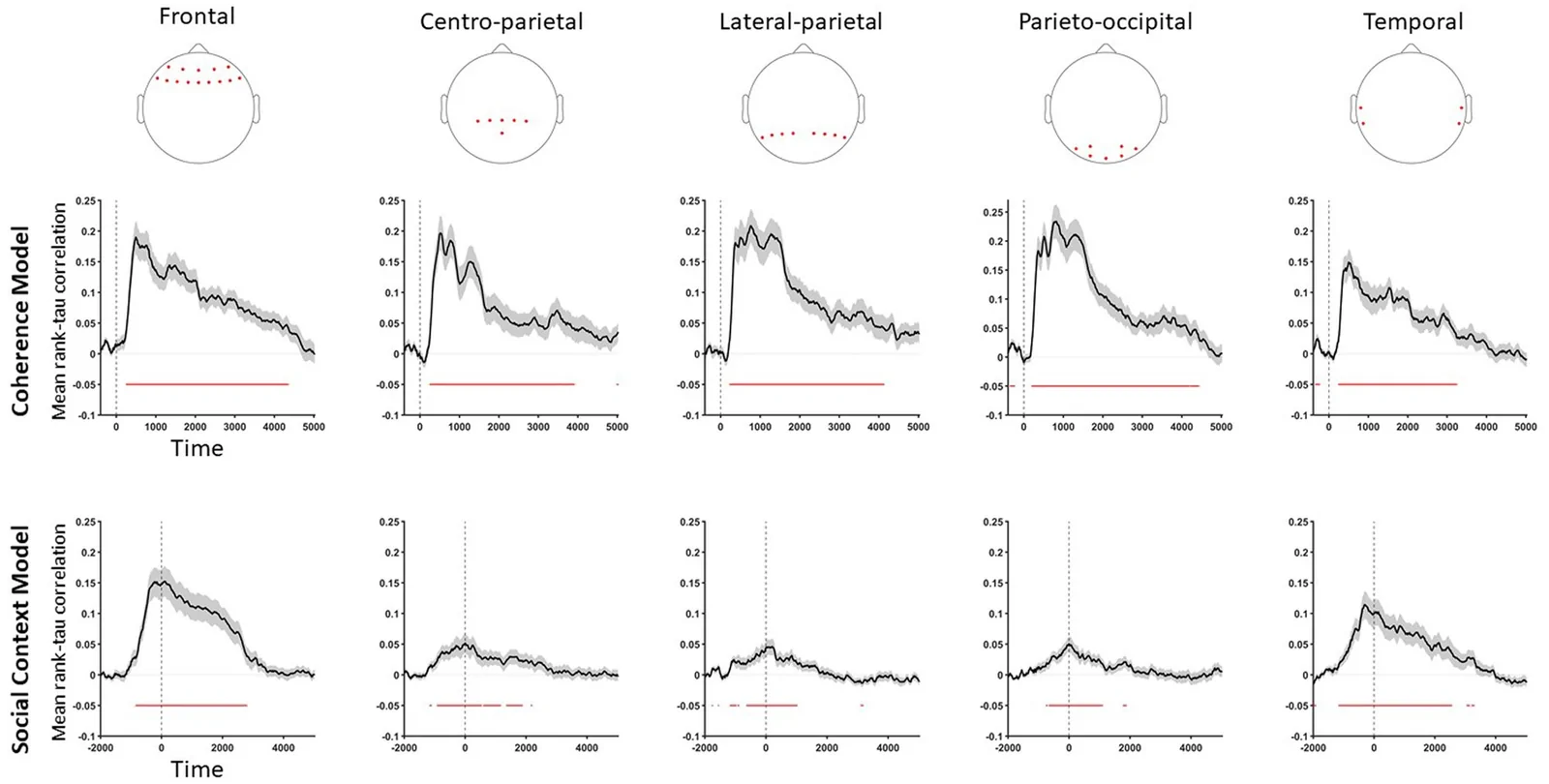
Searchlight RSA. Top row: electrode groups used in each searchlight iteration (left-to-right: frontal, centro-parietal, lateral-parietal, parieto-occipital, temporal). Middle and bottom rows: mean correlations between model RDMs (coherence and condition, respectively) and neural RDMs for each cluster. Red horizontal bars mark significant time windows; dashed vertical lines mark motion onset. Shaded bands indicate SE.

We incorporated a searchlight approach into our analysis and conducted RSA on five different electrode clusters (frontal, centro-parietal, lateral-parietal, temporal, and parieto-occipital). The results showed that both coherence and social context representations were observed across almost the entire sensor space. However, the representation of the social context was rather limited in time and differed temporarily in different clusters compared to coherence representation. Figure 8 presents the RSA results for coherence and condition models for each electrode ROI. Temporal and statistical details of all the observed clusters can be found in the Supplementary Material.

In summary, the behavioral results confirmed our pre-registered hypotheses that participants did not show any difference in accuracy, reaction times, and confidence ratings between public and private trials. In contrast, we did not observe the expected effect in metacognitive efficiency between social and private trials. Similarly, ERP results of CPP also showed no difference between conditions. Yet, our pupil findings showed that in social trials, participants showed higher pupil diameter during the post-stimulus time window. Finally, we have conducted representational similarity analysis, which showed that both motion coherence and social context are encoded in EEG signals. However, the time windows of these representations were different.

## Discussion

Perception is often shared with others, but not necessarily coordinated. We may see the same objects as others without interacting or following their attention, yet publicly perceived objects differ from those seen only by us. They can become points of coordination or competition, justifying a form of social vigilance. This study investigated whether, and to what extent, social vigilance affects human perceptual processing at neural and behavioral levels. Our design allowed us to disentangle the effects of social vigilance from joint attention and mere social presence. Instead of using avatars or on-screen cues to imply presence, we used an actual in-person presence, building on recent calls for ecologically valid research in social neuroscience (Redcay and Schilbach 2019, Fan et al. 2021).

Behaviorally, we replicated the established relationship between sensory evidence—i.e. motion coherence—and accuracy, reaction time, and confidence, supporting our paradigm’s reliability. As hypothesized, results showed no significant difference between private vs. public perception in accuracy, reaction time, confidence, or metacognitive judgments. These null results are theoretically informative: they suggest that social vigilance, as operationalized here, does not translate into strategic changes in decision performance when stakes are low.

As reviewed, prior studies on shared awareness in perception report mixed findings. For example, perspective-taking can facilitate or interfere with performance depending on context (Samson et al. 2010), and learning others’ perceptual decisions often induces behavioral changes (Ogawa et al. 2023). Null results are underreported, but still raise the question: where does our design diverge? First, the second agent did not interact or appear to perform the task. Participants received no input or cue about any “decision” from the confederate. To minimize confounds like performance anxiety or perceived evaluation (i.e. social threats, see Park et al. 2023), we withheld feedback and presented the confederate as a neutral peer. Our paradigm excluded coordination mechanisms like gaze-following, joint attention, or cooperation. No competitive or collaborative framing was used. These design features isolated co-perception, minimizing other social influences. Our lab model captured the experience of sitting next to someone on public transport, aware they might glimpse at what you’re reading. There is no overt “behavior,” but a social interaction still takes place. Our behavioral results suggest the setup neither boosted nor impaired performance.

Our EEG results align with prior literature on perceptual decision-making. Stimulus-locked ERPs revealed a robust CPP scaling with sensory evidence strength, consistent with CPP reflecting evidence accumulation (O’Connell et al. 2012, Kelly and O’Connell 2013). Response-locked analyses showed CPP converged to a consistent amplitude across trials, regardless of stimulus strength—supporting its role in reaching a decision threshold per accumulation-to-bound models (Twomey et al. 2016). These findings affirm CPP as a neural signature of accumulation reaching a bound at response execution, and show EEG-based decision signals generalize across contexts, validating our paradigm.

Analysis of the CPP revealed no significant differences between public and private conditions. Given our sample size and robust design, this null result likely reflects true insensitivity to minimal social context. Although CPP is reliably linked to accumulation, its amplitude remained unchanged by co-perception. Other studies showing social effects often involved social feedback or perspective-taking (Germar et al. 2014, Zanesco et al. 2019a, 2019b, Chen et al. 2024; see Parker and Ramsey 2024). The absence of an ERP effect may indicate that co-perception, without explicit interaction or evaluation, does not modulate early neural decision signals. Alternatively, this helps define the boundary conditions for social influences on cognition: some neural signals like CPP may be robust to minimal context.

Univariate ERP amplitude analyses may miss subtle neural differences. Early work using multivariate pattern analysis (MVPA) and classifiers (Haynes and Rees 2006, Kamitani and Tong 2005) revealed that perceptual states could be decoded from distributed activity even when univariate methods failed. Later, representational similarity analysis (RSA) provided a framework to assess neural representational structure (Kriegeskorte et al. 2008, Nili et al. 2014). These advances show condition differences can exist without amplitude effects. Confirming that ERP amplitudes did not distinguish public and private trials, we applied RSA to examine whether co-perception was nonetheless encoded in distributed EEG activity.

We first validated RSA by assessing how motion coherence was represented across EEG sensor space. Two significant temporal clusters emerged. The first, between 500 and 2000 ms post-stimulus, matched the CPP component and reflected evidence accumulation. The second, around 3500–4000 ms, occurred after accumulation, likely indexing post-decisional processes like confidence or response execution (Pleskac and Busemeyer 2010, Navajas et al. 2016). These findings confirm RSA captures coherence-related neural dynamics, supporting its use for probing co-perception encoding.

RSA revealed that, unlike ERP amplitudes, distributed EEG activity encoded differences between public and private conditions. A significant temporal cluster emerged before stimulus onset—during the fixation period when condition cues were presented. This suggests co-perception is proactively represented, not just an outcome of processing. This aligns with findings that social context can shape preparatory states (Graziano and Kastner 2011). We interpret this pre-stimulus cluster as anticipatory coding of social relevance. There are three non-exclusive mechanisms which may explain the finding: (i) attentional readiness, configuring preparatory control that biases early processing at stimulus arrival; (ii) task-state/context updating, whereby the cue updates and maintains an internal model of “public” vs. “private” until onset; and (iii) anticipation of social relevance. Because our design was neutral and non-evaluative (no feedback or explicit judgment), interpretations centered on evaluation apprehension are unlikely; however, potential expectancy of social relevance in (iii) cannot be entirely excluded without further orthogonal manipulations. Accordingly, the functional specificity of this pre-stimulus RSA effect remains inferential. Without orthogonal manipulations disentangling expectancy, social vigilance, and attentional readiness, our interpretation cannot yet be considered definitive and should be regarded as a tentative interpretation that remains to be tested directly.

In relation to the predictive coding framework, we suggest that the public/private cue can be seen as a prior that configures processing via top-down modulation of preparatory states. This interpretive link clarifies that our effect reflects a task-state/internal-model representation rather than a change in evidence accumulation per se in this minimal setup. Consistent with predictive-coding perspectives on social action and emotion, such priors are expected to bias feedback pathways that prepare action-perception circuits; critically, these anticipatory signals should translate into behavioral outputs when the observed actions matter (e.g. competition, audience, coordination; Keysers et al. 2024). In our minimal co-visibility setup, we indeed found no changes in DDM bias or drift, but these models predict stronger behavioral expressions when co-visibility is consequential, providing a concrete target for future manipulations.

In this light, the pre-stimulus RSA cluster is best read as social vigilance rather than social facilitation or evaluation apprehension: our design lacked feedback or explicit judgment, and the DDM showed no context differences in starting point or drift. Furthermore, Searchlight RSA showed the strongest condition differences in frontal and temporal regions (sensor space; coarse localization without source reconstruction), suggesting involvement of higher-order social and attentional processes (Lau and Rosenthal 2011). This anticipatory representation may reflect an internal model of shared perception (Frith and Frith 1999, Friston and Frith 2015). Frontal and temporal involvement further supports the idea that co-perception recruits social-cognitive resources, such as theory of mind (Schilbach et al. 2013). Thus, while co-perception doesn’t affect early perceptual encoding or accumulation, it is encoded pre-stimulus and in distributed activity, possibly influencing attention or motivation in situations beyond the minimal conditions tested here.

Pupil size analysis showed that co-perception modulated arousal, reinforcing the concept of social vigilance. While post-decisional pupil size was unchanged, stimulus-locked responses were larger in public trials, especially within 1000 ms of stimulus onset. Given the typical 1-second delay in pupil dynamics (Joshi et al. 2016, de Gee et al. 2014), this likely reflects early perceptual or attentional effects. Pupil dilation tracks noradrenergic activity and arousal (Aston-Jones and Cohen 2005), and is elevated under social evaluation (Müller-Pinzler et al. 2015). Under our social vigilance account, the larger stimulus-locked pupil dilation on public trials indexes arousal-linked readiness that accompanies the anticipatory encoding of the social context, consistent with noradrenergic engagement that can modulate processing gain. Beyond overt evaluation cont, work on passive-viewing (Ricou et al. 2024) also shows that pupil diameter scales with the social and motion content of stimuli, indicating sensitivity to social relevance per se rather than mere task engagement. However, it is important to acknowledge that here the observed social context effect was rather small, even though it was statistically robust. This magnitude aligns with “context-only” social manipulations in pupillometry and is smaller than effects observed in response to explicit social/emotional visual content (e.g. larger dilation for faces/avatars vs. objects; Ricou et al. 2024) or under acute stress, where supportive touch reduces pupil reactivity (Graff et al. 2019). Methodological reviews also emphasize that pupil effect sizes are strongly constrained by luminance and stimulus-control considerations; when these are tightly equated, observed differences are typically modest (Mathôt 2018). Taken together, our findings suggest that co-perception, even without interaction, increases physiological arousal—potentially preparing observers for shared relevance without altering decisions.

Given this arousal effect, a key question is whether the RSA-observed neural signature reflects social vigilance or other task factors. As described in Methods, private and public trials were matched on trial count, motion direction, coherence levels, and presentation fields, ensuring RSA differences reflect internal awareness of co-perception. However, the null behavioral results call for caution in our interpretation. In this minimal co-visibility setup, DDM fits showed no differences between public and private trials in starting-point (bias) or drift-rate parameters, suggesting that anticipatory coding did not translate into decision-criterion or evidence-accumulation changes here. We predict such shifts would emerge when co-visibility is behaviorally consequential—for example, under competition (comparative performance), audience effects, or coordination demands. A 2 × 2 design crossing co-visibility (present/absent) with evaluation pressure or incentive framing (competition/cooperation/neutral) would test for selective interactions in pre-stimulus coding, pupil-linked readiness, and decision parameters. Combined with pupil results, current findings suggest social vigilance is encoded both physiologically and neurally—modulating pre-stimulus brain states without changing behavior. These covert responses might reflect preconscious readiness for social relevance, supporting our interpretation of “social vigilance.” At the same time, given the small magnitude of the pupil effect and the fact that the RSA effects, although medium-to-large in within-subject terms, still index relatively subtle modulations in neural representational geometry, these findings should therefore be understood as subtle changes rather than large shifts in neural or autonomic state. Their functional interpretation should be treated with caution.

Even when there’s no reason to adapt responses; no competition, cooperation, or communication, social vigilance persists as a pre-stimulus readiness. This contrasts with studies involving coordination or incentives where social context affects sensitivity or speed (Seow and Fleming 2019). Still, neural signals show that merely knowing others see the same stimulus can modulate anticipatory neural states. Pupil size analysis supports this: after controlling for coherence and confidence, we found a modest but reliable increase in public trials. Since pupil dilation is automatic and linked to arousal, this suggests co-perception triggers involuntary readiness for co-visibility. Our study contributes to theoretical accounts of subtle social context shaping arousal and cognitive preparedness, even without behavioral effects. Such covert markers could aid understanding social salience processing in clinical groups, like those with social anxiety or autism, where atypical responses to social presence are observed (e.g. Odriozola et al. 2016).

### Conclusion and limitations

These findings of social vigilance—sensitivity to the fact that an object is seen by someone else—are novel and offer avenues for future work. First, we show that minimal social sharing is neurally represented even without behavioral effects, inviting future studies to explore when and how such sharing influences behavior.Second, our neural findings are constrained by EEG’s limited spatial resolution. Our temporally focused RSA approach cannot link results to specific networks or brain regions. Future studies using MEG or fMRI could provide spatially detailed insights. Third, this study focused on visual perception, though perceptual experience is multisensory. Expanding to other modalities could test the generalizability of social vigilance. Future research might examine whether social vigilance extends to more private senses like gustation or olfaction.

Overall, our paradigm targets a minimal, neutral form of co-perception (dyadic co-visibility without interaction or evaluation), which constrains direct generalization to richer group contexts. We predict that the observed pre-stimulus neural representation will scale with social relevance and more strongly manifest in behavior when co-visibility is consequential (e.g. audience pressure, competition, coordination). Future studies can vary in group size, audience composition, and shared-outcome contingencies, and use source-resolved methods (e.g. MEG/fMRI) to further investigate network level correlates. Practically, co-visibility should be measured or controlled when it is a potential confound (e.g. shielded displays; randomized audience presence; reporting shared-visibility procedures) and—contingent on replication and validation—the combined pre-stimulus RSA and stimulus-locked pupillometry signature could complement assessments of altered social-salience processing in clinical populations. New experimental designs afforded by smartphone apps and displays that inherently distinguish the public and private view could also be incorporated profitably in this research. These limitations and highlighted future directions suggest opportunities for replication and expansion, deepening our understanding of social vigilance in perception.

## Supplementary Material

nsag009_Supplementary_Data

## Data Availability

All preprocessed behavioral, EEG and pupillometry datasets of the present work as well as code snippets for statistical analysis and visualizations can be openly accessed via: https://doi.org/10.5281/zenodo.17177034.
