# Supplementary material for: Covert neural and autonomic signatures of shared perception
Source: Soc Cogn Affect Neurosci. 2026 Feb 17;21(1):nsag009. doi: 10.1093/scan/nsag009 (PMC13034546; doi:10.1093/scan/nsag009)
Supplement: nsag009_Supplementary_Data [file nsag009_supplementary_data.pdf]

Supplementary Material

Pilot Study

A pilot study with the same setup and procedure with the main study (details explained in later part of this document) was conducted with 9 human subjects. Behavioral and ERP results from this pilot used in sample size estimation for the main study. Also, we have added pupil size recordings for the main study and change the levels of motion coherence used (added 25.6% motion coherence to the existing 4 levels: 3.2, 6.4, 12.8 and 51.2)

Behavioral Results

No difference in accuracy, RT and confidence ratings between public and private perception was found. We have found a trend in metacognitive efficiency between public and private trials, participants showed higher metacognitive efficiency in public trials.

For Peer Review

Table S1. Accuracy by Motion Coherence and Condition

	**Accuracy**				
Predictors	*Odds Ratios*	*std. Error*	*CI*	*Statistic*	*p*
Intercept	0.9976	0.1000	0.8197 – 1.2140	-0.0244	0.981
Condition	1.0054	0.0688	0.8793 – 1.1496	0.0785	0.937
Coherence	1.0823	0.0046	1.0734 – 1.0914	18.6242	**<0.001**
ICC	0.0174				
$N_{subject}$	9				
Observations	5042				
Marginal R^2 / Conditional R^2	0.415 / 0.425				
AIC	5013.602				

Table S2. Confidence by Motion Coherence and Condition

	**Accuracy**				
Predictors	*Odds Ratios*	*std. Error*	*CI*	*Statistic*	*p*
Intercept	0.2266	0.0666	0.1274 – 0.4030	-5.0531	**<0.001**
Condition	1.0486	0.0754	0.9107 – 1.2073	0.6595	0.510
Coherence	1.0885	0.0029	1.0828 – 1.0943	31.4779	**<0.001**
ICC	0.1839				
$N_{subject}$	9				
Observations	5042				
Marginal R^2 / Conditional R^2	0.404 / 0.513				
AIC	4784.877				

For Peer Review

Table S3. Reaction Time by Motion Coherence and Condition

	**SoC Rating**				
Predictors	*Estimates*	*std. Error*	*CI*	*Statistic*	*p*
Intercept	7.3320	0.1337	7.0699 – 7.5942	54.8291	**<0.001**
Coherence	-0.0111	0.0003	-0.0116 – -0.0105	-40.2513	**<0.001**
Condition	-0.0040	0.0107	-0.0250 – 0.0170	-0.3721	0.710
ICC	0.5247				
$N_{subject}$	9				
Observations	5042				

Marginal R^2 / Conditional R^2	0.133 / 0.588
AIC	4663.891

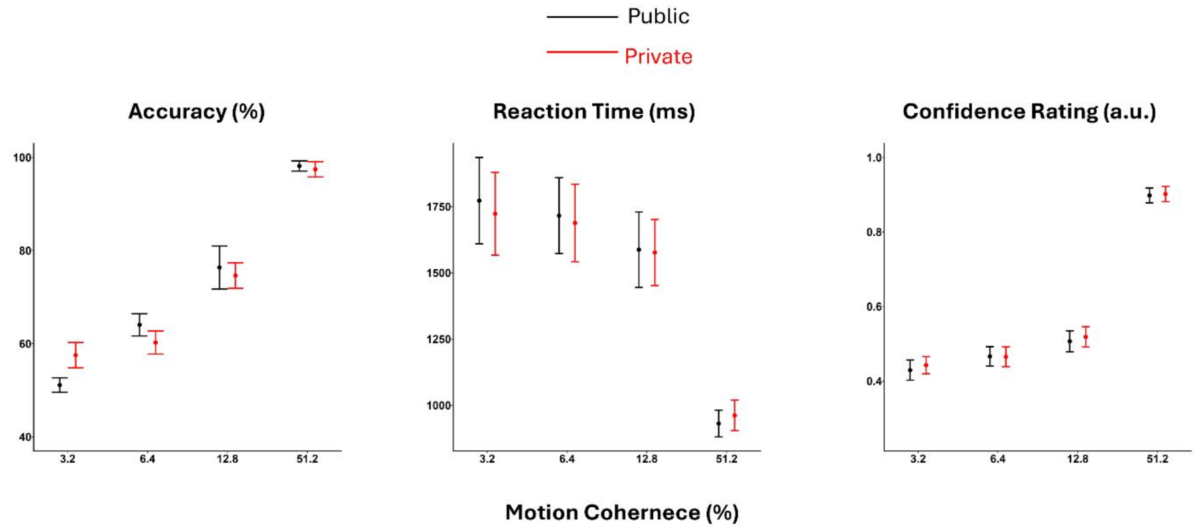

Figure S1. Modulation of accuracy, reaction times and confidence ratings by motion coherence. Colors indicate social context, and error bars show group-level SEs.

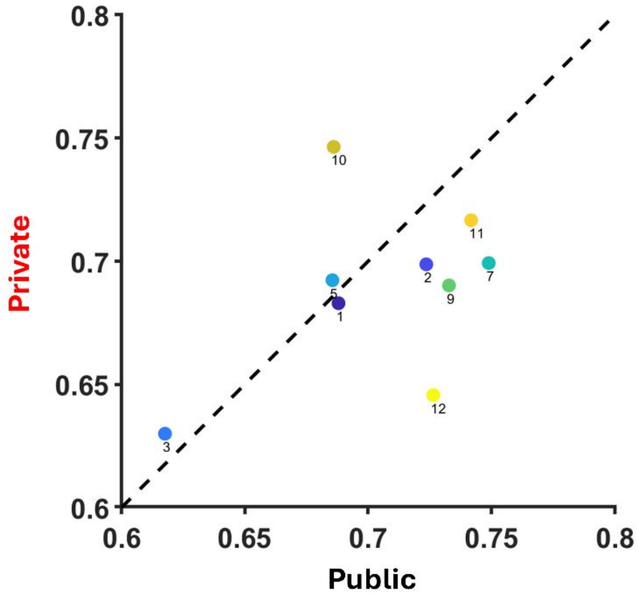

Figure S2. AUCs of ROC analysis for public and private trials per each subject. Each dot represents a unique subject while numbers next to them are unique subject identifiers.

ERP Results

We have found a trend in CPP amplitude analysis between public and private perception trials. Public trials yielded higher CPP amplitude.

Table S4. Mean CPP Amplitude by Motion Coherence and Condition

	CPP Amplitude				
Predictors	*Estimates*	*std. Error*	*CI*	*Statistic*	*p*
Intercept	9.2063	5.4500	-1.4781 – 19.8907	1.6892	0.091
Coherence	0.0983	0.0283	0.0428 – 0.1537	3.4754	**0.001**
Condition	-2.0865	1.0995	-4.2420 – 0.0690	-1.8977	0.058
ICC	0.1338				
$N_{Subject}$	9				
Observations	5093				
Marginal R^2 / Conditional R^2	0.003 / 0.136				
AIC	51875.285				

Sample Size Estimation

Sample size estimation was done on pilot study data, by running mixed-effect regression models on CPP amplitude data, by using motion coherence and social context as fixed factors and subjects as random effects. Then we followed a bootstrapping procedure for sample size estimation by extending the model along the number of subjects and simulating power for each levels of sample size for 1000 times, using SIMR package (Green & MacLeod, 2016).

http://mc.manuscriptcentral.com/scan

For Peer Review

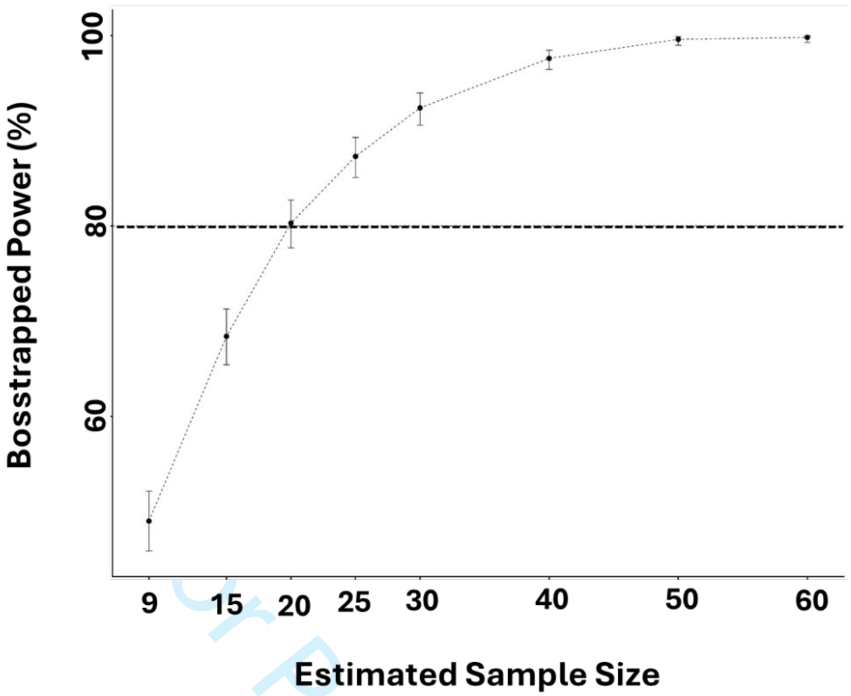

Figure S3. Simulation-based bootstrapped power values for sample size estimation. Estimations in the model were based on experimental condition. Dashed horizontal line show 80% power as our pre-determined cut-off value.

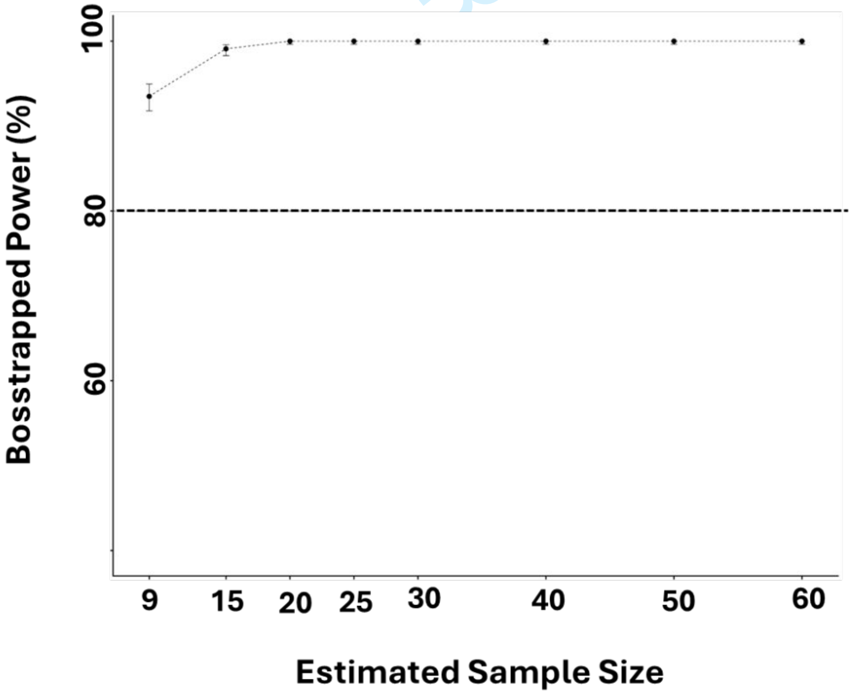

Figure S4. Simulation-based bootstrapped power values for sample size estimation. Estimations in the model were based on motion coherence. Dashed horizontal line show 80% power as our pre-determined cut-off value.

Main Study

Participants

Our initial sample consisted of 34 healthy adults (mean age: 23.88, SD = 4.39, 11 males). The required sample size for the study was determined by a pilot study that we conducted (link to pilot study design, pilot results, sample size estimation, exclusion criteria and other relevant details are available at pre-registration OSF Page). One of the participants performed around chance level (e.g., 50% accuracy) during the warm-up block and had to be excluded from further steps of the experimental sessions. Due to a failure during the data export process, pupillometry data from 4 participants could not be saved. Thus, the final sample consisted of 33 participants with complete behavioral and EEG datasets, for 29 of whom pupillometry data were also available. All participants were right-handed, had no history of neurological or psychiatric disorders, and had normal or corrected-to-normal vision. The study was approved by the ethics committee of the General and Experimental Psychology Department of Ludwig-Maximillian University, Munich. Participants were provided either course credits or 25 Euros in compensation for their time in participating in the study. Recruitment was done by using the University's online research participation platform. Written informed consents for participation were taken before the start of each experimental session, and all types of data from the participants were anonymized from the very beginning.

Procedure

Each participant started the experiment with a warm-up block consisting of 160 trials. Initially, they were presented with blocks of 10 trials from each difficulty level (50 trials in total) to gain an understanding of the overall difficulty of the task. Each participant first received 10 trials with the highest coherence (i.e., the easiest level) and after each 10 trials the difficulty level of the stimulus increased. Once 50 trials were completed in this incremental difficulty fashion, then the task difficulty was randomized across trials until the end of the warm-up block (as it was in the test blocks). During the warm-up block, participants received visual feedback (correct, incorrect) on the screen after each response. Trial-by-trial feedback was not provided during the main experiment. After each block (i.e., 160 trials), participants received overall accuracy for the completed block.

http://mc.manuscriptcentral.com/scan

Once they were ready, the confederate entered the room and was introduced to the participant. The cover story for the confederate was constant across recordings. The confederate was introduced as another student from the University's recruitment system and between the two of them, the confederate was randomly assigned to the role of "observer" whereas the subjects were randomly assigned to the "participant" role. Instructions about the task were mentioned once again in the presence of the confederate, and they were also instructed that while the participants were performing the task, they were not allowed to communicate with each other, neither verbally nor non-verbally. Once the instructions were clear, participants were free to start the block. After each block, their average accuracy was displayed on the screen. They were allowed to take breaks between the blocks to ensure minimal mental load and avoid fatigue.

Once they were ready, the confederate entered the room and was introduced to the participant. The cover story for the confederate was constant across recordings. The confederate was introduced as another student from the University's recruitment system and between the two of them, the confederate was randomly assigned to the role of "observer" whereas the subjects were randomly assigned to the "participant" role. Instructions about the task were mentioned once again in the presence of the confederate, and they were also instructed that while the participants were performing the task, they were not allowed to communicate with each other, neither verbally nor non-verbally. Once the instructions were clear, participants were free to start the block. After each block, their average accuracy was displayed on the screen. They were allowed to take breaks between the blocks to ensure minimal mental load and avoid fatigue.

Survey Data

Participants responded to 2 questionnaires after they had completed the decision-making task. They responded to the English versions of the Social Phobia Inventory (SPIN) (Connor et al., 2000) and Cognitive Flexibility Inventory (CFI) (Dennis and Vander Wal, 2010). SPIN is a self-report measure of social anxiety disorder symptoms, with 17 items evaluated via a 5-point Likert-type scale whereas CFI is a self-report measure of cognitive flexibility, including 20 items that are evaluated on a 7-point Likert-type scale. Cumulative scores from each survey were computed by simple summation of the responses to each item. Higher scores in SPIN indicate higher levels of self-reported social anxiety, whereas higher scores in CFI indicate higher levels of self-reported cognitive flexibility.

Behavioral Results

Regression Tables for Behavioral Models

Table S5. Accuracy by Motion Coherence and Condition

For Peer Review

Predictors	*Odds Ratios*	*std. Error*	*CI*	*Statistic*	*p*
Intercept	1.2389	0.1144	1.0338 – 1.4846	2.3201	0.020
Condition	1.0338	0.0433	0.9523 – 1.1223	0.7939	0.427
Coherence	1.1298	0.0037	1.1225 – 1.1371	36.8703	<0.001
ICC	0.0652				
$N_{Subject}$	33				
Observations	20227				
Marginal R^2 / Conditional R^2	0.565 / 0.593				
AIC	14247.088				

For Peer Review

Table S6. Confidence by Motion Coherence and Condition

Predictors	*Odds Ratios*	*std. Error*	*CI*	*Statistic*	*p*
Intercept	0.1546	0.0306	0.1049 – 0.2278	-9.4326	<0.001
Condition	1.0633	0.0403	0.9871 – 1.1453	1.6172	0.106
Coherence	1.1227	0.0020	1.1187 – 1.1267	63.4388	<0.001
ICC	0.2743				
$N_{Subject}$	33				
Observations	20227				
Marginal R^2 / Conditional R^2	0.475 / 0.619				
AIC	17352.123				

Table S7. Reaction Time (log-transformed) by Motion Coherence and Condition

Predictors	*Estimates*	*std. Error*	*CI*	*Statistic*	*p*
Intercept	7.2427	0.0346	7.1749 – 7.3105	209.3480	<0.001
Coherence	-0.0140	0.0002	-0.0143 – -0.0136	-87.5108	<0.001
Condition	-0.0097	0.0056	-0.0206 – 0.0013	-1.7344	0.083
ICC	0.1969				
$N_{Subject}$	33				
Observations	20227				
Marginal R^2 / Conditional R^2	0.233 / 0.384				
AIC	20236.342				

114

115 **EEG Results – Regression for CPP Amplitude**

Table S8. Mean CPP Amplitude by Motion Coherence

Predictors	*Estimates*	*std. Error*	*CI*	*Statistic*	*p*
Intercept	2.2524	0.4321	1.4053 – 3.0994	5.2120	<0.001
Coherence	0.0326	0.0025	0.0278 – 0.0375	13.2126	<0.001
Condition	0.0277	0.0862	-0.1413 – 0.1966	0.3209	0.748
ICC	0.1344				
$N_{Subjects}$	33				
Observations	18976				
Marginal R^2 / Conditional R^2	0.008 / 0.141				

For Peer Review

AIC	121610.876

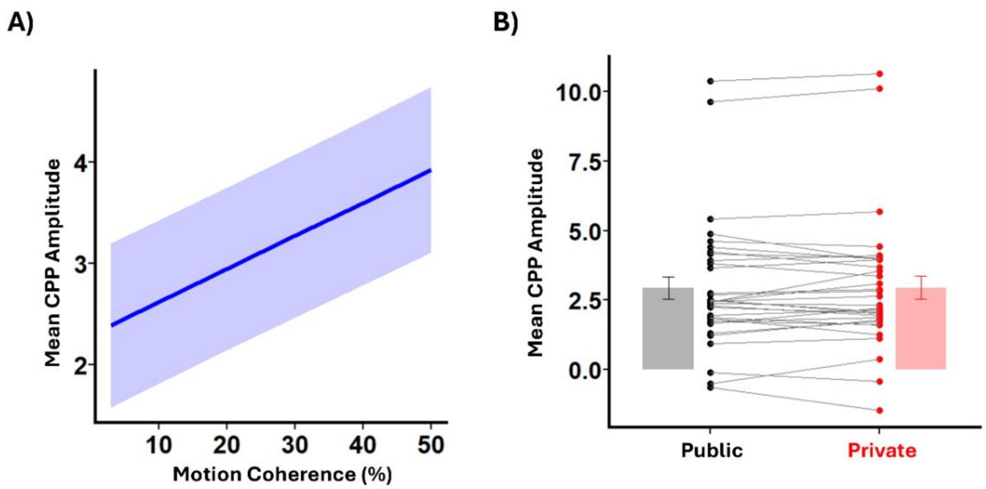

Figure S5. Mean CPP amplitude by motion coherence and social condition. Panel A shows results from regression model for motion coherence. Panel B shows mean (bars) and individual values (dots) for CPP amplitude for each condition. Error bars show 1 SEs.

Pupil Results

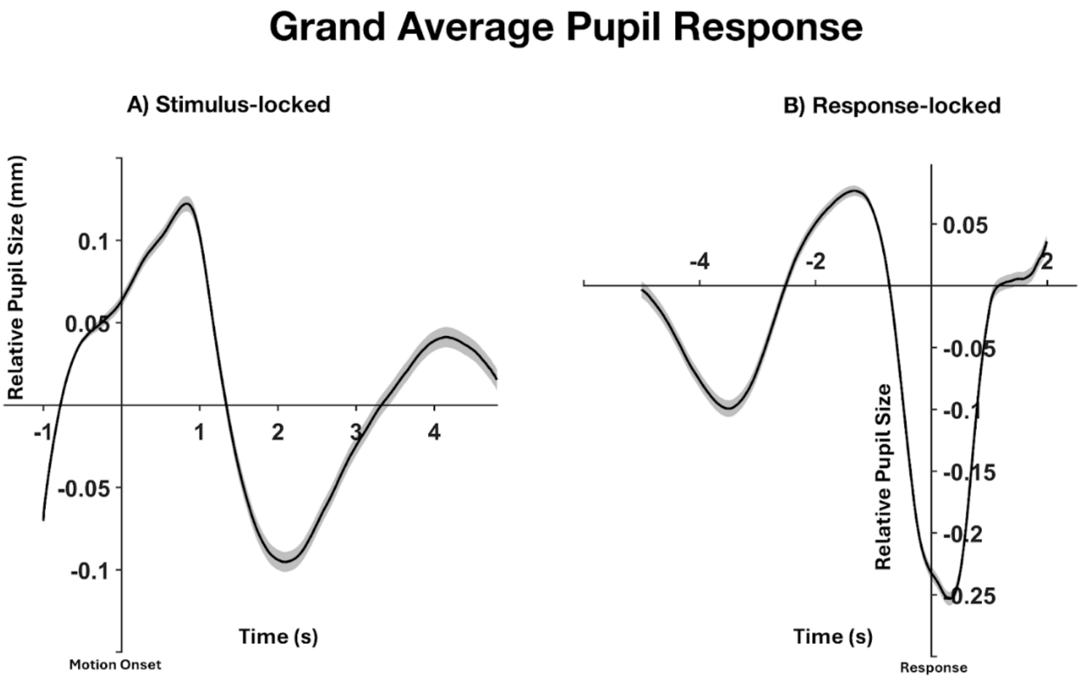

Figure S6. Grand average pupil responses across experimental conditions and subjects. On the left, stimulus-locked pupil response. On the right, response-locked pupil size change. Shaded areas around the signals show SE.

Regression Tables

Table S9. Post-Response (1-second) Pupil Size by Motion Coherence, Condition and Confidence

Predictors	*Estimates*	*std. Error*	*CI*	*Statistic*	*p*
Intercept	-0.3382	0.0199	-0.3772 – -0.2991	-16.9801	<0.001
Coherence	0.0358	0.0074	0.0214 – 0.0503	4.8510	<0.001
Condition	0.0085	0.0074	-0.0060 – 0.0230	1.1538	0.249
Confidence	0.1082	0.0083	0.0918 – 0.1245	12.9876	<0.001
ICC	0.1411				
$N_{Subjects}$	29				
Observations	18108				
Marginal R^2 / Conditional R^2	0.027 / 0.164				
AIC	1112.010				

For Peer Review

Whole Sensor Space RSA – Cluster Permutation Results

Table S10. ***Coherence Model***

Cluster Nr.	Cluster Mass	Mean T Value	Peak T Value	*p*	df
1	208.1977339	13.45266523	0.0022	32	[-3000, -2954] ms
2	830.9497233	3.154234005	0	32	[-2362, -1740] ms
3	212.328494	2.732343695	0.0016	32	[-1734, -1556] ms
4	1413.439534	4.452719661	0	32	[-1546, -626] ms
5	349.5681101	5.923642918	0	32	[-612, -422] ms
6	13921.55767	10.5732201	0	32	[258, 4642] ms
7	176.1393339	2.582618336	0.019	32	[4662, 4808] ms

Table S11. ***Condition Model***

Cluster Nr.	Cluster Mass	Mean T Value	Peak T Value	*p*	df	Time Range
1	10264.50623	5.563418012	8.587738105	0.000999001	32	[-916, 2772] ms

Searchlight RSA – Cluster Permutation Results

Table S12. ***Frontal – Coherence***

Cluster Nr.	Cluster Mass	Mean T Value	Peak T Value	*p*	df	Time Range
1	66.16718008	2.205572669	2.336959107	0.001	32	[-2296, -2238] ms
2	85.40412816	2.18984944	2.28441387	0	32	[-2042, -1966] ms
3	87.64764645	2.137747474	2.224096961	0	32	[-1918, -1838] ms
4	845.011665	3.083984179	3.842670467	0	32	[-1542, -996] ms
5	245.2867498	3.607158085	5.019755422	0	32	[-600, -466] ms
6	9914.501109	4.831628221	7.617857407	0	32	[252, 4354] ms

Table S13. ***Frontal – Condition***

Cluster Nr.	Cluster Mass	Mean T Value	Peak T Value	*p*	df	Time Range
1	8910.865068	4.893391031	6.349255237	0	32	[-840, 2800] ms

Table S14. ***Centro-Parietal– Coherence***

Cluster Nr.	Cluster Mass	Mean T Value	Peak T Value	*p*	df	Time Range
1	86.0843584	2.207291241	2.383210882	0.002	32	[-2476, -2400] ms
2	156.3045016	2.40468464	2.678313976	0	32	[-2114, -1986] ms
3	78.9606494	2.256018554	2.415915244	0.005	32	[-1604, -1536] ms
4	6963.624556	3.801105107	7.036576411	0	32	[250, 3912] ms

For Peer Review

5	243.6879116	2.256369552	2.415105951	0	32	[4990, 5204] ms

Table S15. ***Centro-Parietal– Condition***

Cluster Nr.	Cluster Mass	Mean T Value	Peak T Value	*p*	df	Time Range
1	82.7102744	2.363150697	2.598122554	0.000999001	32	[-2590, -2522] ms
2	53.5855288	2.232730367	2.323714006	0.000999001	32	[-1158, -1112] ms
3	2058.77736	2.827990879	3.458887424	0.000999001	32	[-916, 538] ms
4	150.7534636	2.153620909	2.263868297	0.000999001	32	[604, 742] ms
5	459.8589295	2.254210439	2.475654989	0.000999001	32	[760, 1166] ms
6	470.5780191	2.341184175	2.497807932	0.000999001	32	[1360, 1760] ms
7	54.25434285	2.086705494	2.145598422	0.000999001	32	[1786, 1836] ms
8	28.92083235	2.065773739	2.086811858	0.000999001	32	[1852, 1878] ms
9	29.09485753	2.078204109	2.108382453	0.000999001	32	[2164, 2190] ms

Table S16. ***Lateral-Parietal– Coherence***

Cluster Nr.	Cluster Mass	Mean T Value	Peak T Value	*p*	df	Time Range
1	9201.645669	4.713957822	7.703389671	0	32	[228, 4130] ms
2	1251.529343	2.559364709	3.219194485	0	32	[5022, 5998] ms

Table S17. ***Lateral-Parietal– Condition***

Cluster Nr.	Cluster Mass	Mean T Value	Peak T Value	*p*	df	Time Range
1	47.56464803	2.26498324	2.345250704	0.000999001	32	[-2702, -2662] ms
2	53.3877642	2.224490175	2.337869618	0.000999001	32	[-2344, -2298] ms

For Peer Review

3	68.23170569	2.27439019	2.496178502	0.000999001	32	[-2224, -2166] ms
4	14.87053029	2.12436147	2.170437972	0.000999001	32	[-1758, -1746] ms
5	12.46162455	2.076937425	2.098718142	0.000999001	32	[-1556, -1546] ms
6	280.3961926	2.832284773	3.426136712	0.000999001	32	[-1172, -976] ms
7	35.2692617	2.074662453	2.109735374	0.000999001	32	[-918, -886] ms
8	2248.121477	2.728302763	3.683290808	0.000999001	32	[-628, 1018] ms
9	16.71482006	2.089352507	2.118339006	0.000999001	32	[3116, 3130] ms
10	46.94501406	2.133864275	2.181693958	0.000999001	32	[3136, 3178] ms
11	121.6716143	2.172707398	2.328593055	0.000999001	32	[5280, 5390] ms
12	31.56656261	2.104437508	2.162853824	0.000999001	32	[5398, 5426] ms

Table S18. ***Parieto-Occipital– Coherence***

Cluster Nr.	Cluster Mass	Mean T Value	Peak T Value	*p*	df	Time Range
1	167.6941671	2.942002932	3.352230512	0.032	32	[-344, -232] ms
2	9995.307469	5.005161477	8.444970784	0	32	[206, 4198] ms
3	261.1897142	2.535822467	2.880788418	0	32	[4228, 4432] ms

Table S19. ***Parieto-Occipital– Condition***

Cluster Nr.	Cluster Mass	Mean T Value	Peak T Value	*p*	df	Time Range
1	34.1201304	2.13250815	2.231764217	0.000999001	32	[-744, -714] ms
2	2669.448141	3.040373737	4.17880358	0.000999001	32	[-654, 1100] ms

For Peer Review

3	113.7381781	2.187272656	2.260936563	0.000999001	32	[1778, 1880] ms

Table S20. ***Temporal– Coherence***

Cluster Nr.	Cluster Mass	Mean T Value	Peak T Value	*p*	df	Time Range
1	149.6470014	2.720854571	3.08378331	0	32	[-344, -236] ms
2	6712.29838	4.465933719	7.198577332	0	32	[244, 3248] ms

Table S21. ***Temporal– Condition***

Cluster Nr.	Cluster Mass	Mean T Value	Peak T Value	*p*	df	Time Range
1	231.2565526	3.125088549	3.601581318	0.000999001	32	[-2352, -2206] ms
2	205.0611207	2.888184798	3.979428232	0.000999001	32	[-2056, -1916] ms
3	6739.415883	3.625290954	5.356158571	0.000999001	32	[-1158, 2558] ms
4	96.74593595	2.149909688	2.19499894	0.000999001	32	[3054, 3142] ms
5	86.5059729	2.109901778	2.186666514	0.000999001	32	[3216, 3296] ms

For Peer Review

Correlation Analysis of Measured and Computed Variables

For each variable, we have computed the ratio between public and private trials per subject. For instance, we computed mean reaction times for public and private trials per subject, then calculated the ratio between them. Then, we have correlated these ratios between variables (see Table S22) to check even though there were no group level differences whether there are any individual differences that can be seen in the datasets we have. CPP, Stimulus-locked pupil size, response-locked pupil size ratios correspond to mean values of these metrics during the reported time-windows. Threshold and slope ratios correspond to values obtained from psychometric fitting. Coherence RSA and Condition RSA are correlation values for each subject, taken from the time point where the correlation between model RDMs and brain RDMs were maximum. ACC (accuracy), RT, Conf ratios are derived from raw behavioral data from each subject for each condition and AUC Ratio is obtained from metacognitive efficiency scores. Finally, Social Anxiety and Cog. Flex scores are total survey scores of participants.

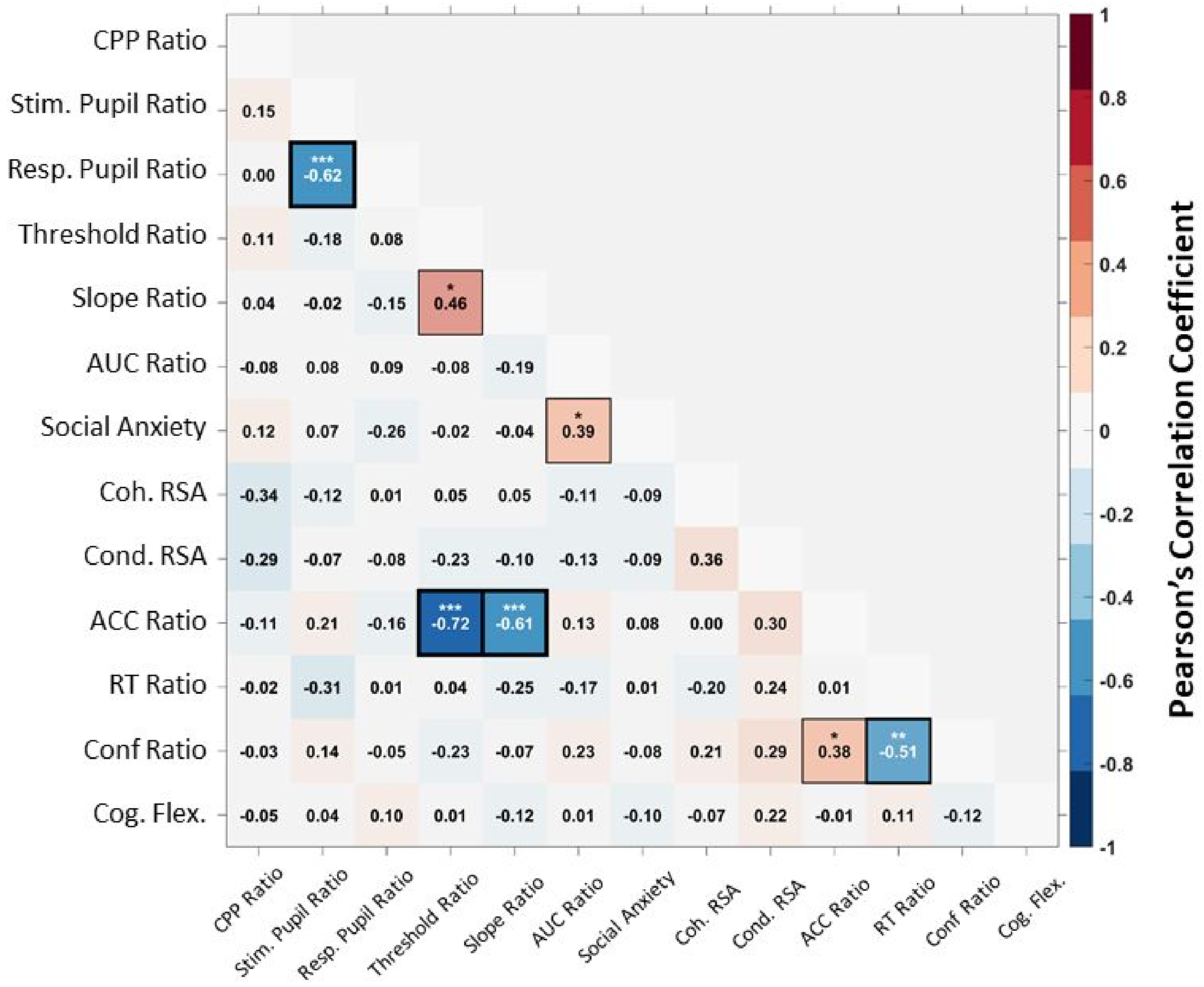

Figure S9. Heatmap of pairwise correlations between measured and computed variables. Transparency (alpha values) of the cells are adjusted based on obtained p values of the correlations. Alpha 1.00 for p <0.001, alpha 0.80 for p < 0.01, alpha 0.60 for p < 0.05 and alpha 0.25 for non-significant results. * *p < 0.05,* ** *p < 0.01,* *** *p < 0.001*

Table S22. Pairwise Correlations between Measured and Computed Variables

	1	2	3	4	5	6	7	8	9	10	11	12	13
**1. CPP Ratio**	**1.00**												
**2. Stim. Pupil Ratio**	.15	**1.00**											
**3. Resp. Pupil Ratio**	.00	**-.62*****	**1.00**										
**4. Threshold Ratio**	.11	-.18	.08	**1.00**									
**5. Slope Ratio**	.04	-.02	-.15	**.46***	**1.00**								
**6. AUC Ratio**	-.08	.08	.09	-.08	-.19	**1.00**							
**7. Social Anxiety**	.12	.07	-.26	-.02	-.04	**.39***	**1.00**						
**8. Coh. RSA**	-.34	-.12	.01	.05	.05	-.11	-.09	**1.00**					
**9. Cond. RSA**	-.29	-.07	-.08	-.23	-.10	-.13	-.09	.36	**1.00**				
**10. ACC Ratio**	-.11	.21	-.16	**-.72*****	**-.61*****	.13	.08	.00	.30	**1.00**			
**11. RT Ratio**	-.02	-.31	.01	.04	-.25	-.17	.01	-.20	.24	.01	**1.00**		
**12. Conf Ratio**	-.03	.14	-.05	-.23	-.07	.23	-.08	.21	.29	**.38***	**-.51****	**1.00**	
**13. Cog. Flex.**	-.05	.04	.10	.01	-.12	.01	-.10	-.07	.22	-.01	.11	-.12	**1.00**

* $p < 0.05$, ** $p < 0.01$, *** $p < 0.001$

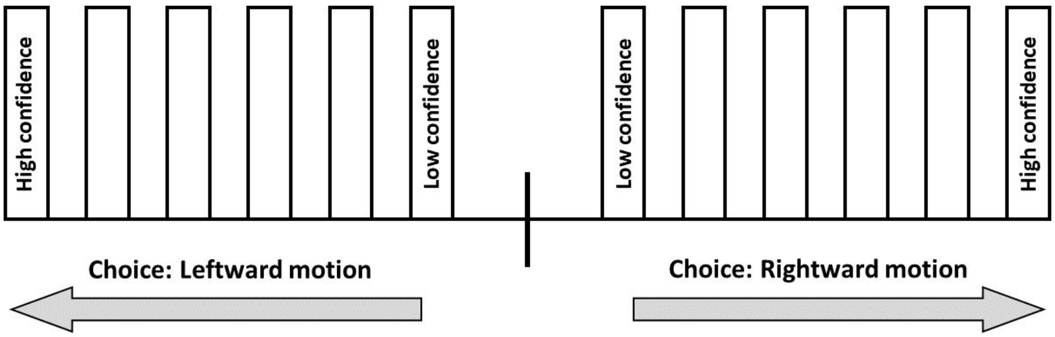

Figure S10. Schematic representation of the confidence rating interface during the task. In each trial, participants used this rating scale to simultaneously report their binary choice of motion direction and their confidence in their choice. The interface appeared neutrally in the experiment, no confidence rating bar was pre-selected and there were no verbal indicators of confidence. The cursor's location was in the middle of the scale (filled, black vertical line in the middle of the scale), and by moving the mouse towards right or left, participants were able to select their confidence ratings per each trial. Once they are on the corresponding bar to report their confidence, they finalise it by a left-click. They were familiarized with this method of reporting choice and confidence during the warm-up block. In the real interface, they did not see the texts or the arrows on the figure above, they are only here for illustrative purposes.

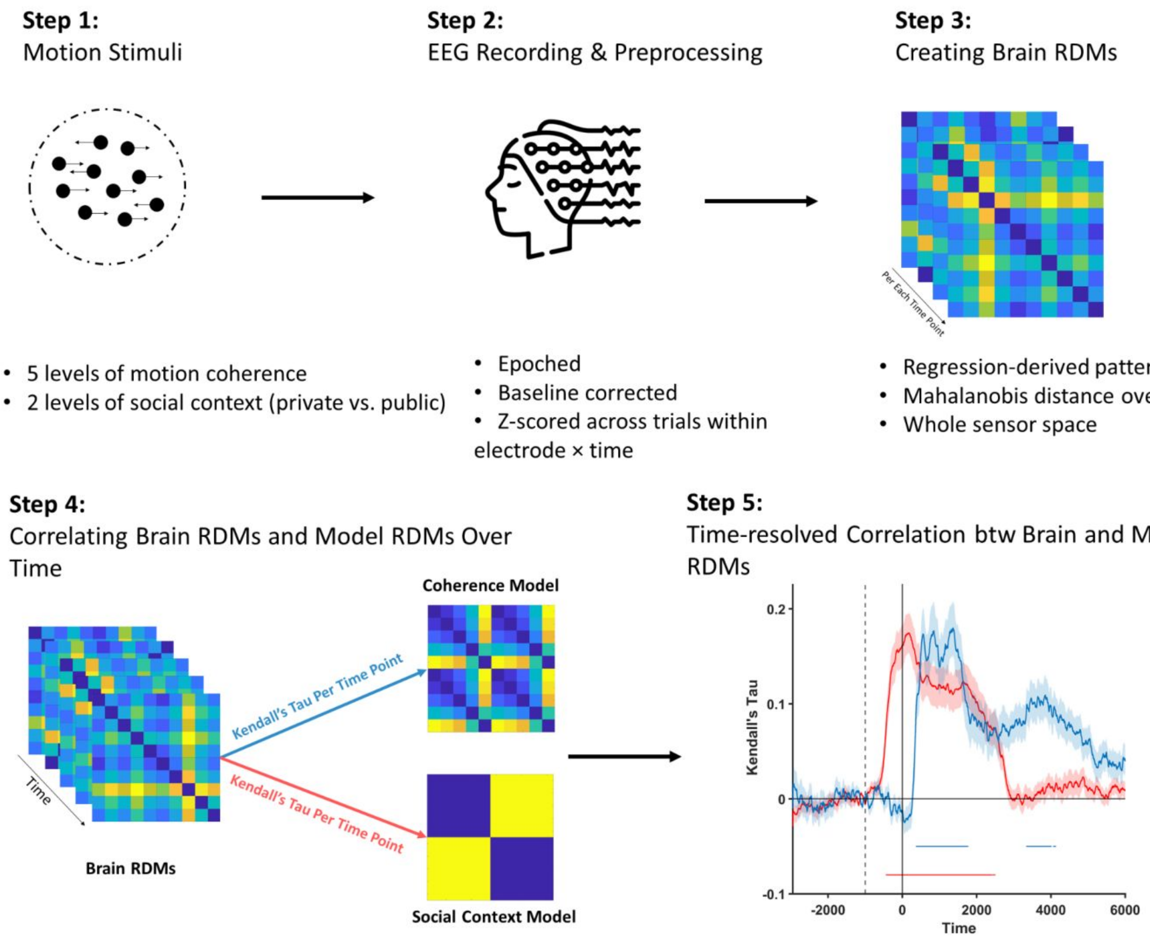

Figure S11. Schematic of the EEG representational similarity analysis (RSA) pipeline.

Step 1 – Stimuli & conditions: Motion stimuli were presented at five coherence levels (3.2–51.2%) under two social contexts (private vs. public). Step 2 – Preprocessing: EEG was epoched around stimulus onset, baseline-corrected, and z-scored across trials for each electrode × time point. Step 3 – Brain RDMs: Using whole-sensor (all electrodes) activity, condition-specific patterns were obtained from a trial-wise regression (intercept excluded). At each time point, pairwise Mahalanobis distances among the 10 conditions (5 coherence × 2 context) formed a 10×10 Brain representational dissimilarity matrix (RDM). Step 4 – Model RDMs: Two theoretical RDMs captured expected structure: (i) a coherence model encoding absolute differences in motion coherence and replicated across contexts; and (ii) a social-context model assigning zeros to within-context pairs and ones to between-context pairs. Step 5 – Model–Brain comparison: For each time point, the Brain RDM was correlated with each Model RDM using Kendall's tau-a (rank correlation suited to tied values), yielding time-resolved model–brain similarity without invoking any univariate ERP assumptions.

Table S23. Mean Pupil Size During Context-Sensitive Time Window by Condition and Coherence

Predictors	*Estimates*	*std. Error*	*t*	*df*	*CI*	*p*
Intercept	0.0678	0.0121	5.63	31	[0.0432, 0.0924]	< .001
Condition	-0.0117	0.0026	-4.56	18276	[-0.0168, -0.0067]	< .001
Motion	-0.0019	0.0001	-26.36	18276	[-0.0021, -0.0018]	< .001
ICC	0.118					
N (subjects)	29					
Observations	18305					
Marginal R^2 / Conditional R^2	0.033 / 0.147					
AIC	-11912.558					

For Peer Review

References

Connor, K. M., Davidson, J. R., Churchill, L. E., Sherwood, A., Weisler, R. H., & Foa, E. (2000). Psychometric properties of the Social Phobia Inventory (SPIN): New self-rating scale. The British Journal of Psychiatry, 176(4), 379-386.

Dennis, J. P., & Vander Wal, J. S. (2010). The Cognitive Flexibility Inventory: Instrument Development and Estimates of Reliability and Validity. Cognitive Therapy and Research, 34(3), 241–253. https://doi.org/10.1007/s10608-009-9276-4

Green, P., & MacLeod, C. J. (2016). SIMR: An R package for power analysis of generalized linear mixed models by simulation. Methods in Ecology and Evolution, 7(4), 493–498. https://doi.org/10.1111/2041-210X.12504

For Peer Review
